# Therapeutic potential of paeoniflorin in atherosclerosis: A cellular action and mechanism-based perspective

**DOI:** 10.3389/fimmu.2022.1072007

**Published:** 2022-12-21

**Authors:** Wei Yu, Iqra Ilyas, Xuerui Hu, Suowen Xu, Hui Yu

**Affiliations:** ^1^ School of Materials Science and Engineering, Hefei University of Technology, Hefei, Anhui, China; ^2^ Center for Drug Research and Development, Anhui Renovo Pharmaceutical Co., Ltd, Center for Drug Research and Development, Hefei, Anhui, China; ^3^ Department of Endocrinology, The First Affiliated Hospital of USTC, Division of Life Sciences and Medicine, University of Science and Technology of China, Hefei, Anhui, China; ^4^ School of Pharmacy, Anhui University of Chinese Medicine, Hefei, Anhui, China; ^5^ School of Materials Science and Engineering, Tianjin Key Laboratory of Materials Laminating Fabrication and Interfacial Controlling Technology, Hebei University of Technology, Tianjin, China

**Keywords:** atherosclerosis, cardiovascular disease, paeoniflorin, inflammation, immunity

## Abstract

Epidemiological studies have shown that the incidence, prevalence and mortality of atherosclerotic cardiovascular disease (ASCVD) are increasing globally. Atherosclerosis is characterized as a chronic inflammatory disease which involves inflammation and immune dysfunction. *P. lactiflora* Pall. is a plant origin traditional medicine that has been widely used for the treatment of various diseases for more than a millennium in China, Japan and Korean. Paeoniflorin is a bioactive monomer extracted from *P. lactiflora* Pall. with anti-atherosclerosis effects. In this article, we comprehensively reviewed the potential therapeutic effects and molecular mechanism whereby paeoniflorin protects against atherosclerosis from the unique angle of inflammation and immune-related pathway dysfunction in vascular endothelial cells, smooth muscle cells, monocytes, macrophages, platelets and mast cells. Paeoniflorin, with multiple protective effects in atherosclerosis, has the potential to be used as a promising therapeutic agent for the treatment of atherosclerosis and its complications. We conclude with a detailed discussion of the challenges and future perspective of paeoniflorin in translational cardiovascular medicine.

## 1 Introduction

It is estimated that cardiovascular diseases (CVD), consisting of >40% of global non-communicable disease (NCD) mortality, causes 17.9 million deaths per year and is projected to exceed 23.6 million deaths per year by 2030 (1). The dominant cause of CVD is atherosclerosis (2). The classical theory of atherosclerosis suggests that the entire progression of the lesion can be divided into three stages: initiation, progression and acute events (3). The development of atherosclerosis commences with endothelial dysfunction caused by endothelial cell damage, which in turn allows monocytes in the blood to be recruited at the site of injury. Monocytes then enter the damaged endothelium and are induced to differentiate into macrophages. These macrophages and the smooth muscle cells migrate into the intima, phagocytose lipids until they evolve into foam cells. The accumulation and apoptosis of foam cells further form the core of the atheromatous plaque, leading to stenosis and sclerosis of the arterial lumen (4). Atherosclerotic plaques are at very high risk of uncertainty; they may remain clinically asymptomatic for several decades, but sometimes these fragile plaques can become unstable and eventually rupture, which in turn can be life-threatening (5).

Inflammation and immunity-related mechanisms play a critical role in the initiation and progression of atherosclerosis. For example, inflammation of endothelial cells and macrophages, T-cell infiltration within atherosclerotic plaques, activation of Th17 cells, etc. (6). The current first-line therapy for CVD including atherosclerosis is the use of statins (7). The main mechanism is based on the hypolipidemic ability and anti-inflammatory effect of statins to prevent and treat atherosclerosis (8). Despite the widespread use and effectiveness of statins, there is increasing evidence that long-term use of statins may result in many serious adverse effects, the most serious of which is rhabdomyolysis (9).

The discovery of new drugs from natural products has become a new approach to discovering the treatment of CVD including atherosclerosis. Some extracts or single units of natural drugs have shown enormous potential for the therapy of atherosclerosis, such as paeonol (10). As an ornamental and medicinal plant widely distributed around the world, *P. lactiflora* is receiving increasing attention in the research and development of natural drugs. In China and North Asia, *P. lactiflora* is considered as one of the most well-known flowering plants and was cultivated as an ornamental plant throughout the Xia, Shang and Zhou dynasties (11). Its roots are believed to have great medicinal properties for a long time, mainly used for the treatment of pain, inflammation and immune disorders (12).Paeonia is divided into two categories: white peony (*Paeonia alba*) and red peony (*Paeonia lactiflora*), and the medicinal part is the dried root (13). As a Chinese traditional remedy, it has been used in China, Japan and Korean Peninsula for over a thousand years and has good safety and effectiveness (14). The most central active ingredient is paeoniflorin (C_23_H_28_O_11_). In the following section, we will discuss in detail the pharmacological effects and mechanism of action of paeoniflorin in preventing atherosclerosis, focusing on inflammation and immunity-related effects and mechanisms.

## 2 Paeoniflorin

### 2.1 Botanical origin of paeoniflorin

The genus *Paeonia* has 52 species, mainly located in temperate regions of Europe, Asia, and North America (Plant List Database, http://www.theplantlist.org/). China is an important region for the origin, evolution, progression and diversification of the genus. The genus *Paeonia* is further classified into three sections, namely the Sect. *Moutan*, the Sect. *Onaepia* and the Sect. *Paeonia*. The Sect. *Moutan* is the most protologous taxon in the genus, with 11 species, all of them are shrubs or subshrubs, and native to China. There are two species of the Sect. *Onaepia*, both are herbaceous perennials and distributed only in western North America. The Sect. *Paeonia* is a relatively young and evolved taxon in the genus, and also the largest section, with about 22 species, all of which are herbaceous perennials (Plant List Database, http://www.theplantlist.org/).

**Figure 1:**
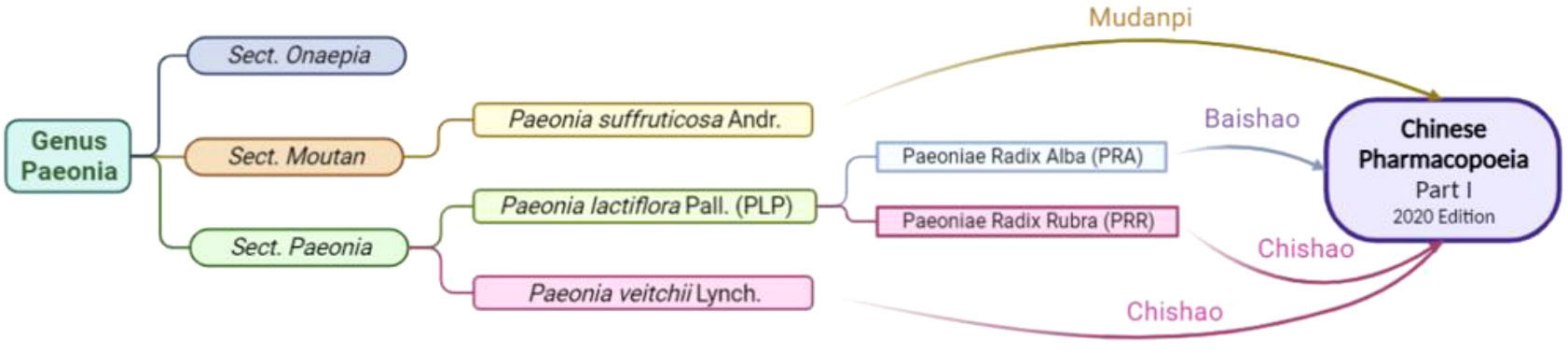
Botanical origin and inclusion in the Chinese Pharmacopoeia of *Paeonia.* Created in BioRender.com.

**Figure 2:**
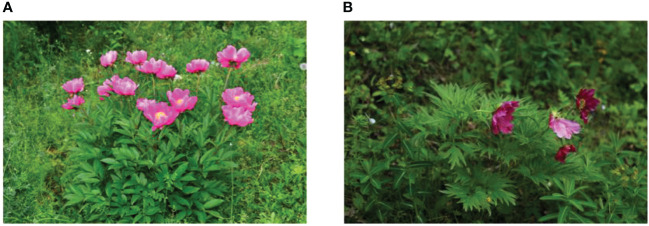
Whole plant of *Paeonia.* The above-ground portion of *Paeonia lactiflora* Pall **(A)**; *Paeonia veitchii* Lynch **(B)**.

**Figure 3:**
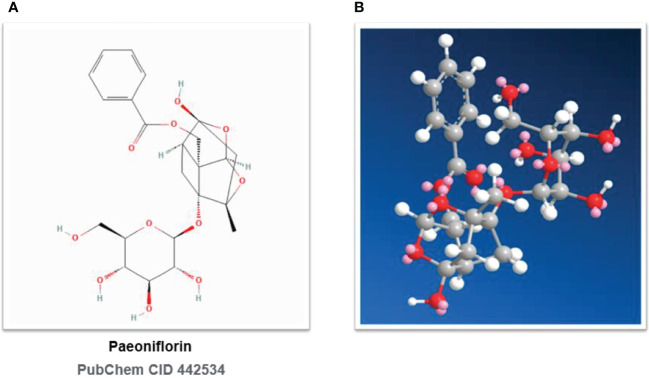
Chemical structures of paeoniflorin. **(A)** Paeoniflorin: 2D Chemical Structural Formula. **(B)** Paeoniflorin: 3D Chemical Structural Formula. PubChem CID 442534.

Three plant sources are included in the Chinese Pharmacopoeia (Part I, 2020 edition): *Paeonia suffruticosa* Andr. of the Sect. *Moutan* (distributed in southwestern and central China), *Paeonia lactiflora* Pall. of the Sect. *Paeonia* (distributed in Eurasia and northwestern Africa), and *Paeonia veitchii* Lynch. (In the western Sichuan and Qinghai-Tibet Plateau of China). In the Chinese Pharmacopoeia, *Paeonia lactiflora Pall.* or noted as *P. lactiflora* Pall. (PLP, also called Shao Yao) is further subdivided into Paeoniae Radix Alba. (PRA, also called baishao or white peony) (15) and Paeoniae Radix Rubra. (PRR, also called chishao or red peony). *Paeonia veitchii* Lynch. (PVL) is classified as red peony (16) in the Chinese Pharmacopoeia. Please refer to **Figures 1**, **2**. For more information on Sect. Moutan, please refer to the reference (10). Specifically, the PLP first appeared in written records in the earliest Chinese pharmacological work<神农本草经> (Shen nong Ben Cao Jing) in Eastern Han Dynasty, 25-220 AD. Tao Hongjing first distinguished it into PRA and PRR in <本草经集注> (Collected Notes on the Materia Medica) in Liang Dynasty, 502-557 A.D. The PRA flowers are white and mostly cultivated, while the PRR is red and mostly wild, and the medicinal parts of both are dried roots (17). In traditional Chinese medical theory, PLP is not used in combination with veratrum root (14). Paeoniflorin (C_23_H_28_O_11_), a water-soluble triterpene glycoside, is the major bioactive ingredient of PLP (18). The chemical structure formula and 3D model of paeoniflorin are shown in **Figure 3**.

The relevant quality test standards for paeoniflorin are detailed in the Chinese Pharmacopoeia (Part I, 2020 edition), while paeoniflorin is approved as the only test index for quality control of PRA and PRR. The Chinese Pharmacopoeia provides for content determination according to high-performance liquid chromatography (HPLC) (General Rule 0512), in which the content of paeoniflorin in PRA shall not be less than 1.2% and the content of paeoniflorin in PRR shall not be less than 1.5%.

### 2.2 Basic physicochemical properties of paeoniflorin

Paeoniflorin, [(1R,2S,3R,5R,6R,8S)-6-hydroxy-8-methyl-3-[(2S,3R,4S,5S,6R)-3,4,5-trihydroxy-6-(hydroxymethyl)oxan-2-yl]oxy-9,10- dioxatetracyclo[4.3.1.02,5.03,8]decan-2-yl]methyl benzoate, PubChem CID No. 442534, Molecular Formula: C_23_H_28_O_11_, Molecular Weight: 480.5, Density: 1.6 ± 0.1 g/cm^3^, Boiling Point: 683.3 ± 55.0°C at 760 mmHg, Flash point: 235.9 ± 25.0°C, Vapor pressure: 0.0 ± 2.2 mmHg at 25°C, Refractive index: 1.683, Polar Surface Area (PSA): 164.

The solubility and oil-water partition coefficient of paeoniflorin in phosphate buffer solution with various pH values had certain effects on the solubility and oil-water partition coefficient, and the trends of solubility and oil-water partition coefficient were almost the same. In the range of pH 2.0-5.0, the solubility and oil-water partition coefficient of paeoniflorin increased with the rise of pH, and in the range of pH 5.0-6.8, the solubility and oil-water partition coefficient decreased with the increase of pH. The solubility and oil-water partition coefficient values of paeoniflorin were greatest in phosphate buffer solution at pH 5.0. Some data (19) showed that the solubility of paeoniflorin ranged from 43.31 to 50.57 mg/ml and the logP (octanol/water partition coefficient) was -0.98 to -0.47. This suggests that paeoniflorin is stable under acidic conditions, and it is possible that paeoniflorin may mainly exists in the molecular form under acidic conditions. Thus, under acidic conditions, the solubility and oil-water partition coefficient values of paeoniflorin are relatively large, while under over-acidic or alkaline conditions, the molecular structure of paeoniflorin was destroyed and existed mainly in ionic form.

### 2.3 Pharmacokinetic properties of paeoniflorin

Pharmacokinetic studies are an essential and crucial part for realizing the transition from laboratory to clinical application. In addition to helping to evaluate the efficacy and potential toxic actions of a medication, it also allows the exploration of interactions between different components. pharmacokinetic studies of paeoniflorin were first reported in 1985 (20). In the last few years, several advanced assays have been applied to the pharmacokinetic study of paeoniflorin for more precise determination and analysis. In the Caco-2 cell model, the transport rate of paeoniflorin was low (21), and this low permeability greatly affects the bioavailability of paeoniflorin. In the rat intestinal perfusion model, paeoniflorin was absorbed in all intestinal segments, but the absorption rate was low and there was no statistically significant difference among the four segments (22). Paeoniflorin is hydrophilic, mainly absorbed by passive diffusion, with the first-pass effect, poor oral absorption, and bioavailability of approximately 3% to 4% (23).

After absorption by enema in rats, paeoniflorin was quickly and extensively distributed in heart, liver, spleen, lung, kidney, brain, stomach and intestine, mainly in stomach (33.66 mg/ml), followed by intestine (19.57 mg/ml) and heart (11.70 mg/ml). The concentration of paeoniflorin in all tissues except liver and stomach peaked at 10 min, after which the levels of paeoniflorin decreased with time (24). In addition, paeoniflorin can cross the blood-brain barrier (BBB), but does not easily penetrate the BBB (25). The half-life (t½) of paeoniflorin is similar in rats and humans (26). Several pharmacokinetic parameters were measured in a clinical study in Chinese volunteers and data showed that the mean half-life (t½) after a single intravenous injection of 18.3 mg, 35.8 mg, or 54.1 mg of paeoniflorin was 1.9 h, 1.9 h, or 1.8 h in males and females, respectively. The clearance (CL) of paeoniflorin was 10.4 L/h, 11.3 L/h, and 11.2 L/h respectively; and the apparent distribution volume ranged from 16.8-18.1. Maximum plasma concentration (Cmax) reached 402.2-1081.0 ng/mL when the dose was increased from 18.3-54.1 mg/mL (27).

Paeoniflorin is mainly metabolized in the intestine, where intestinal bacteria first convert paeoniflorin to paeonolactone glucoside, which is then further metabolized through several pathways such as deglucose, de benzoyl, and quaternary ring cleavage rearrangement. These pathways gradually convert paeoniflorin into metabolites with smaller relative molecular mass and greater hydrophobicity for better absorption by the intestine (28). The main metabolic pathways of paeoniflorin are hydrolysis of ester bonds and glycosides and binding to glucuronide (29). Absorbed paeoniflorin is mainly excreted through urine, and is rarely excreted in the form of the original drug after oral administration (23).

In the process of drug metabolism described above, the paeoniflorin, as the main bioactive ingredient originating from PLP, may have potent efficacy against atherosclerosis and shows potential and far-reaching medicinal value. In the following sections, we will elaborate protective effects of paeoniflorin against atherosclerosis in both *in vivo* and *in vitro* models. We will first present the effects and mechanisms of paeoniflorin on animal models of atherosclerosis and related vascular diseases, and later on the effects and molecular mechanisms of paeoniflorin on important cell types associated with the development and progression of atherosclerosis.

## 3 Effects of paeoniflorin on experimental atherosclerosis

### 3.1 The role of paeoniflorin in animal models of atherosclerosis

Dyslipidemia is one of the common clinical conditions in the progress of atherosclerosis, and high levels of triglycerides (TG) and total cholesterol (TC) are thought to play a very critical function in the progression of the disease (30). Hyperlipidemia causes two conditions. One is the reduced mobility of red blood cells, and the other is the reduced deformability of red blood cells, which further leads to an increase in overall blood viscosity (31). Simultaneously, the metabolism of cholesterol is also very important. Cholesterol accumulation caused by a high-fat diet is one of the most common causative factors of lipid metabolism disorders and is identified in epidemiology as a high-risk factor for CVD (32). Another point of note is that the idea of atherosclerosis as a chronic inflammatory condition is increasingly being recognized by the academic community, and there is increasing evidence that inflammatory processes play a key role in atherosclerosis (33).

Li et al. (34) successfully constructed an atherosclerosis model using Sprague-Dawley (SD) rats fed with high fat diet. Their results showed that paeoniflorin significantly reduced the expression of inflammatory cytokinesand also showed that paeoniflorin could also significantly reduce TC, TG and LDL-C levels in rat serum, and histological analysis of aorta showed that paeoniflorin treatment significantly improved atherosclerotic symptoms in rats. Wu et al. (35) constructed an animal model of atherosclerosis based on APOE^-/-^ mice and examined the effects of paeoniflorin on plasma levels of TC, TG, free cholesterol (FC) and superoxide dismutase (SOD) in mice. Their results showed that paeoniflorin could dose-dependently elevate serum SOD activity, and significantly reduce TC, TG, and FC in serum. Xu et al. (36) also demonstrated that paeoniflorin significantly reduced serum levels of TC/TG/LDL-C in a dose-dependent way in APOE^-/-^ mice and significantly reduced the ratio of plaque area to aortic lumen area in each segment of the aorta.

The above-mentioned experimental studies demonstrate the therapeutic potential of paeoniflorin in animal models of atherosclerosis.

### 3.2 The role of paeoniflorin in other vascular disease models

Hypertension is a pathological disease that damages the endothelium, triggers cell proliferation, vascular remodeling, apoptosis and enhances cell permeability through increased adhesion molecules. Endothelial damage also inhibits vasodilation and increase vasoconstriction in patients, lead to a vicious cycle to further increase blood pressure and exacerbate the development and progression of atherosclerotic lesions (37). Wu et al. (38) established a rat model of gestational hypertension using Wistar rats, and their study demonstrated that paeoniflorin could significantly reduced serum levels of inflammatory. The protection of endothelium and resistance to inflammation suggest that paeoniflorin can play an essential protective role by blocking the vicious cycle of “endothelial damage - elevated blood pressure - atherosclerosis”.

Diabetes is also closely related to atherosclerosis, which can induce the development of atherosclerosis or could further accelerate its progression (39). Recent studies have shown that fluctuating hyperglycemic states are more likely to lead to a variety of vascular complications than chronic persistent hyperglycemia (40). Accumulating evidence (41) suggests that fluctuating hyperglycemia is more potent than constant hyperglycemia (42), volatile blood glucose levels lead to high oxidative stress (43) and inflammation (44). A study conducted by Wang et al. (45) using SD RATS indicated that paeoniflorin could attenuate fluctuating hyperglycemia-induced vascular endothelial injury by restraining oxidative stress, decreasing inflammatory responses. We also searched for other risk factors closely associated with atherosclerosis, such as smoking, unhealthy diet, obesity and sedentary lifestyle, and did not find a direct pharmacological effect of paeoniflorin on these factors.

These above preclinical evidences are summarized in **Table 1**. The results of these animal models of atherosclerosis and related experiments in other models of vascular disease boost our confidence in research work about paeoniflorin as a potential anti-atherosclerotic drug candidate.

**Table 1:** Anti-atherosclerotic effects-related animal experiments with paeoniflorin.

Model	Dose	Route of Administration	Observer Pharmacological Effects	References
**Sprague-Dawley rat**	10 mg/kg, 20 mg/kg	**p.o.** , daily for 18 weeks	TC ↓ TG↓ LDL-C↓	(34)
	10 mg/kg	**p.o.** , daily for 6 weeks	Inflammatory response ↓	(45)
**APOE ^(-/-)^ mice**	60 mg/kg	**p.o.** , daily for 8 weeks	TC↓ TG↓ FC↓ SOD↑	(35)
	30 mg/kg, 60 mg/kg	**p.o.** , daily for 12 weeks	TC ↓ TG↓ LDL-C↓ Plaque area ↓	(36)
**Wistar rat**	100 mg/kg, 300 mg/kg	**p.o.** , daily for 18 days	Inflammatory response ↓	(38)

↓, down-regulation / suppression.

## 4 The detailed mechanism of action of paeoniflorin in combating atherosclerosis

**Figure 4:**
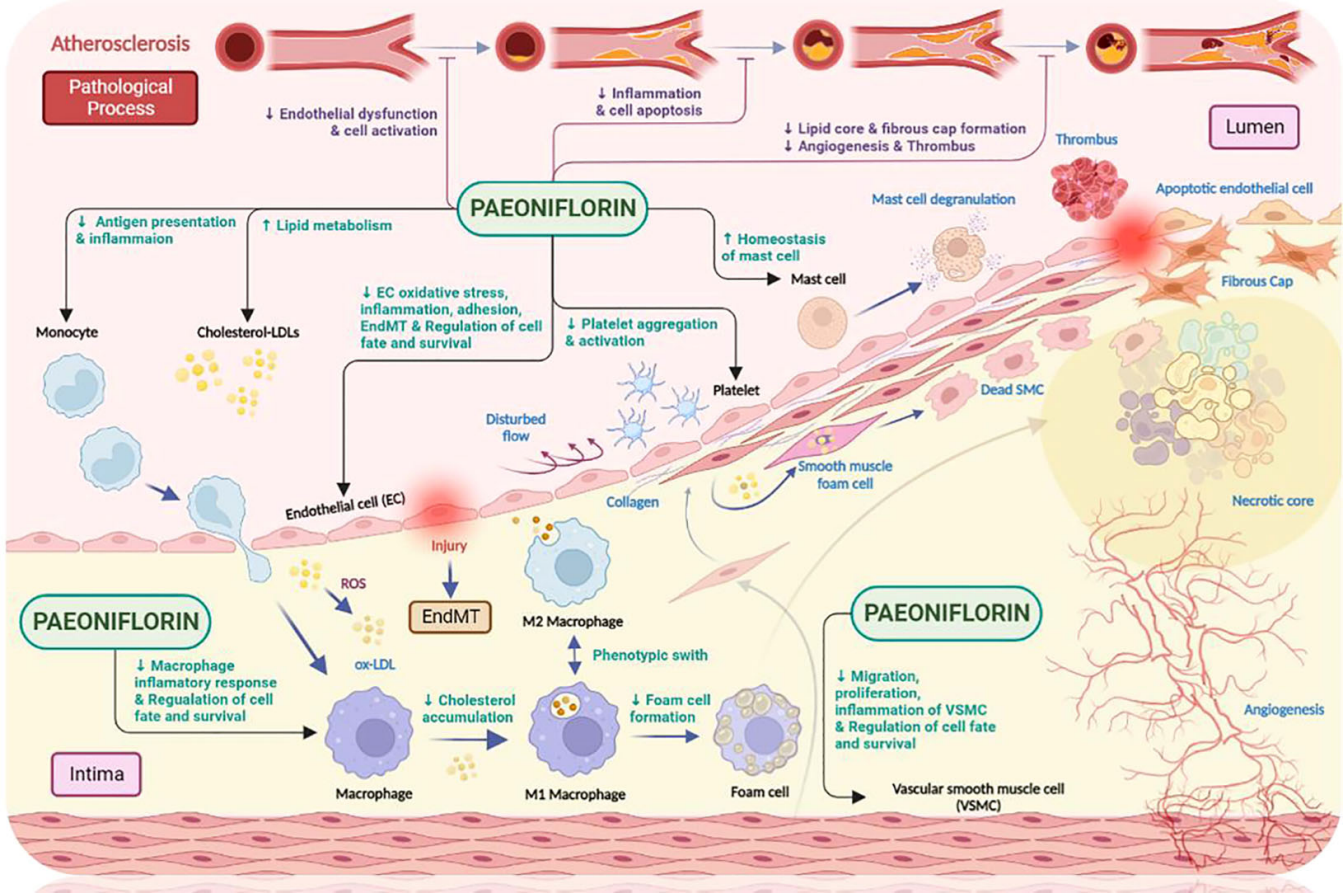
The anti-atherosclerotic effects of paeoniflorin. Paeoniflorin plays a crucial role in the different stages of atherosclerosis development by affecting different cell types (e.g., monocytes, macrophages, mast cells related to innate immunity; endothelial cells, smooth muscle cells and platelets related to process initiation and progression). Paeoniflorin can effectively intervene in the development and progression of atherosclerosis by exploiting different signaling pathways. Created in BioRender.com.

It is well known that atherosclerosis initiation and progression involves the dysfunction of multiple key cell types, the most typical of which include: endothelial cells, smooth muscle cells, monocytes, macrophages, platelets and mast cells. Once these cells become dysfunctional, a series of adverse consequences will occur, including: damage to the endothelium, chronic inflammation, disturbance in lipid metabolism, dysregulation of oxidative stress, changes in the cellular life cycle, proliferation and migration of vascular smooth muscle cells (VSMC) and abnormal platelet aggregation and activation (46). Paeoniflorin has been found to respond favorably to the aforementioned cellular dysfunction and thus exert a protective effect. In the following sections, we will discuss this in detail (**Figure 4**).

### 4.1 Paeoniflorin and endothelial cell function

The vascular endothelium is tightly connected to each other and performs various topological configurations in three dimensions, forming the inner wall of arteries, veins and capillaries and shaping itself into the largest distributive organ in the human body (47). It has been suggested that, as the barrier in the outermost layer of the vasculature, it plays a very vital role in maintenance of vascular homeostasis and health (48). In healthy conditions, the endothelium performs many different functions, including dynamic maintenance of vascular tone, angiogenesis, hemostasis, and provision of antioxidant, anti-inflammatory, and antithrombotic interfaces. Once the vascular endothelium becomes dysfunctional, this is manifested by impaired endothelium-dependent vasodilation, elevated oxidative stress, chronic inflammation, excessive leukocyte adhesion and permeability, endothelial cell senescence and metabolic alterations and endothelial-to-mesenchymal transition. All these mechanisms are involved in the development and progression of atherosclerosis, playing different roles and critical roles at different times (47).

Many external risk factors (such as hyperlipidemia and low/oscillatory shear stress in blood flow) can cause damage to endothelial cells, making their function impaired. During this process, secretion of intercellular adhesion molecule-1 (ICAM-1) and vascular cell adhesion molecule-1 (VCAM-1), as well as a range of other adhesion molecules and/or interleukins, is significantly increased. In parallel, blood monocytes are recruited to the damaged endothelial surface and further differentiate into macrophages. These cells further secrete monocyte chemoattractant protein-1 (MCP-1), which attracts more monocytes and leads to the production of foam cells. The above process is accompanied by the release of inflammatory factors, which in turn leads to a prolonged local inflammatory response in the vasculature, exacerbating the initiation and development of atherosclerosis (49).

**Figure 5:**
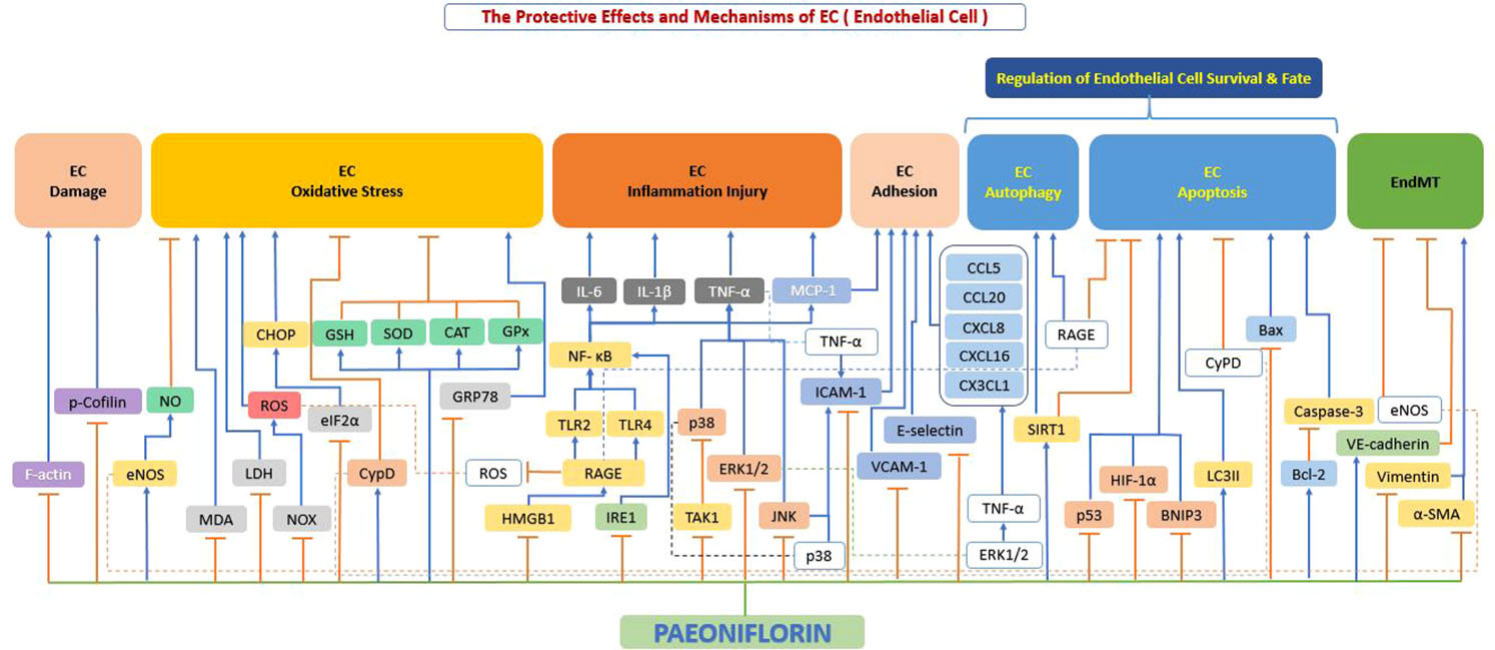
The protective effects and mechanisms of paeoniflorin on endothelial cells. Paeoniflorin can exert protective effects on endothelial cells and thus on atherosclerosis through various mechanisms, which mainly include: inhibition of cell damage, inhibition of oxidative stress, prevention of excessive production of inflammatory factors, improvement of cell adhesion function, regulation of cell autophagy and apoptosis. Bax, Bcl-2-associated X protein; Bcl-2, B-cell lymphoma-2; CAT, catalase; CCL20, c-c chemokine ligand 20; CCL5, c-c chemokine ligand 5; CHOP, C/EBP homologous protein; CX3CL1, c-x-3-c chemokine ligand 1; CXCL16, c-x-c motif chemokine ligand 16; CXCL8, c-x-c motif chemokine ligand 8; CypD, cyclophilin D; eIF2α, eukaryotic initiation factor 2α; eNOS, endothelial nitric oxide synthase; ERK1/2, extracellular signal-regulated kinase 1/2; F-actin, filamentous actin; GPx, glutathione peroxidase; GRP78, glucose-Regulated Protein 78; GSH, glutathione; HIF-1α, hypoxia-inducible factor-1α; HMGB1, high mobility group box 1; ICAM1, intercellular adhesion molecule 1; IL-1β, interleukin 1β; IL-6, interleukin 6; JNK, c-Jun N-terminal kinase; LC3II, light chain 3 II; LDH, lactate dehydrogenase; MCP-1, monocyte chemoattractant protein-1; MDA, malondialdehyde; NF-κB, the nuclear factor-kB; NO, nitrous oxide; NOX, NADPH-oxidase; p38, p38 mitogen-activated protein kinase (MAPK); p53, tumor protein p53 (also known as TP53); p-Cofilin, phosphorylation of cofilin; RAGE, receptor for advanced glycation end products; ROS, reactive oxygen species; Sirt1, sirtuin 1; SOD, superoxide dismutase; TAK1, transforming growth factor beta-activated kinase1; TLR2, toll-like receptor 2; TLR4, toll-like receptor 4; TNF-α, tumor necrosis factor α; VCAM1, vascular cell adhesion molecule 1; VE-cadherin, vascular endothelial (VE)-cadherin; α-SMA, alpha-smooth muscle actin.

Our literature review found that paeoniflorin can exert protective effects on endothelial cells through a variety of mechanisms, thereby exerting effects on atherosclerosis. These mechanisms targeting endothelial cells mainly include suppression of cellular damage, inhibition of oxidative stress, prevention of overproduction of inflammatory factors, improvement of cell adhesion function, regulation of cellular autophagy, and apoptosis (**Figure 5**).

#### 4.1.1 Paeoniflorin inhibited endothelial cell damage

Endothelial cell dysfunction is a predominant characteristic of the initial progression of atherosclerosis (47). Under healthy physiological conditions, endothelial cells are connected to each other forming a selective semi-permeable membrane that serves as a blood-tissue barrier and effectively coordinates the exchange of fluid and macromolecules between the two sides of the vessel wall. However, external stimuli (e.g., inflammation) can cause damage to the morphology and connections of endothelial cells. It results in endothelial cells dysfunction, which can lead to a series of consequences, such as increased vascular permeability, infiltration of plasma macromolecules/leukocytes and edema (50).

It has been reported in the literature that when endothelial cells are subjected to certain adverse stimuli, their actin may be disrupted, which in turn leads to an increase in permeability (51). There are two forms of actin: monomeric (G-actin) and polymeric (F-actin) (52). Among them, F-actin dominates endothelial cell contraction, protrusion and division rearrangement, which mediates endothelial cell injury and leads to increased endothelial permeability (53). Xu et al. (54) showed that paeoniflorin significantly inhibited the expression of cytoskeletal proteins and actin in Lipopolysaccharide (LPS)-stimulated HUVEC and restored stress fibers and cell morphology, thereby suppressing endothelial cell injury.

#### 4.1.2 Paeoniflorin inhibits oxidative stress in endothelial cells

When the body is under oxidative stress, excess of free radicals and intracellular reactive oxygen species (ROS) are released, which can further lead to endothelial dysfunction (55). The mechanism behind this is mainly because excess ROS interferes with the function of eNOS, leading to a decrease in the NO and thus an increase in blood pressure (56). Fu et al. (57) investigated the protective effect of NO on HUVEC, and their results showed that paeoniflorin could dose-dependently increase NO production by increasing eNOS expression, thereby protecting endothelial cells.

At the same time, the release of ROS further exacerbates the oxidation of LDL, leading to the formation of oxidized LDL, all of which can further damage the vascular endothelium. Both lactate dehydrogenase (LDH) and malondialdehyde (MDA) are the most widely used biomarkers when assessing oxidative stress damage as described above (58). To counteract the likely harmful effects of ROS, cells maintain endogenous antioxidant capacity, including the combined action of superoxide dismutase (SOD), glutathione (GSH) and other endogenous antioxidants, which can either directly counteract ROS or indirectly regulate ROS levels (59). Yu et al. (60) displayed that paeoniflorin significantly reduced the formation of ROS, MDA content and LDH leakage levels in endothelial cells and increased the activity of endogenous antioxidants GSH and SOD, effectively counteracting endothelial damage from oxidative stress.

It is well known that the core processes of cellular energy metabolism are regulated by mitochondria, which are the most important place for most ATP production in the body (61). Increased mitochondrial ROS production and progressive respiratory chain dysfunction are associated with atherosclerosis or cardiomyopathy (62). Song et al. (63) study showed that paeoniflorin effectively inhibited AOPP-induced ROS production in HUVECs in a dose-dependent manner. Paeoniflorin also successfully reduced the ratio of damaged mitochondria and effectively restored ATP levels by inhibiting ROS and restoring ATP depletion and mitochondrial dysfunction. Paeoniflorin inhibited RAGE mRNA and Nox2/Nox4 protein expression, reduced the production of ROS, effectively resisted oxidative stress, and significantly improved endothelial cell viability. Jiang et al. (64) indicated that paeoniflorin significantly reduced TBHP-induced excessive intracellular ROS production and restored TBHP-induced mitochondrial depolarization, improved TBHP-induced mitochondrial functional impairment in HUVEC, and restored SOD, CAT, and GPx activities, corroborating the existence of oxidative stress resistance effect of paeoniflorin from the other side.

If we turn our attention to the metabolism of calcium, we will notice that calcium dysregulation leads to a sustained transfer of calcium ions from the endoplasmic reticulum to the mitochondria. It results in protein spatial folding dysfunction and chronic inflammatory calcium overload in mitochondria, followed by induction of apoptosis *via* a Bcl2-dependent mechanism (65). Cyclophilin D (CypD) plays a crucial role in protein folding (66). Walter et al. reported that C/EBP Homologous Protein (CHOP) is recognized as a transcription factor that induces apoptosis in endoplasmic reticulum stress-induced cells (67). Activation of PERK leads to phosphorylation of eIF2α, which is considered to play a primary function in the triggering of CHOP in response to endoplasmic reticulum stress (68). Ou et al. (69) revealed that paeoniflorin pretreatment could reduce H_2_O_2_-mediated oxidative stress levels in endothelial cells by inhibiting intercellular ROS and Ca^2+^ production. Secondly, paeoniflorin pretreatment could reduce H_2_O_2_-mediated mitochondrial dysfunction by activating CypD and promoting CypD expression. Finally, paeoniflorin pretreatment could reduce H_2_O_2_-mediated endoplasmic reticulum stress by blocking the eIF2a- CHOP signaling pathway and reducing H_2_O_2_-induced endoplasmic reticulum stress. Therefore, paeoniflorin pretreatment can reduce the toxicity of oxidative stress to endothelial cells from these three dimensions and protect endothelial cells. A study by Chen et al. (70) also corroborated that paeoniflorin significantly reduced the overproduction of endoplasmic reticulum stress markers (78 kDa glucose regulated protein (GRP78) and CCAAT/CHOP), thereby reversing the ultrastructural abnormalities of the endoplasmic reticulum.

From the above we can know that supplementation with antioxidants may be a promising strategy to prevent oxidative stress damage in endothelial cells. In recent years, natural antioxidants have attracted attention for its effectiveness and low toxicity (71). Our literature review shows that paeoniflorin possesses the potential to be a natural antioxidant that could effectively exert a role against oxidative stress in endothelial cells.

#### 4.1.3 Paeoniflorin inhibits the secretion of inflammatory factors from endothelial cells

Inflammation is an integral part of the pathogenesis of several cardiovascular diseases, including atherosclerosis. Release of inflammatory cytokines, such as TNF-α and IL-6, activates monocytes and induces monocyte adhesion to endothelial cells (72). Subsequently, monocytes are recruited and adhere to the endothelium, where they further compartmentalize into macrophages, thereby exacerbating inflammation and the release of pro-inflammatory substances (73). Bi et al. (74) showed that paeoniflorin notably inhibited the secretion of inflammatory factors (IL-6, TNF-α) and the activation of IκB-α, NF-κB (p65), p38, and JNK in HUVECs, counteracting the inflammatory effects. Chen et al. (70) found that paeoniflorin significantly inhibited the p-IRE1 α upregulation, which is involved in multiple signaling pathways leading to immune activation and inflammation and is a critical step in activating the inflammatory response in the vessel wall (75). Blockage of this pathway could effectively block subsequent responses to the inflammatory process, effectively inhibit IL-6 production and thus attenuate inflammatory damage to endothelial cells.

Lysophosphatidylcholine (LPC) is the major biologically active component of oxidized LDL and is an essential contributor to atherosclerotic activity (76). High mobility group box-1 (HMGB1) stimulates the expression and release of several inflammatory factors such as TNF-α, interleukins, ICAM-1 and selectins (77). The pro-inflammatory effects of HMGB1 are mainly mediated *via* binding to its corresponding receptors, including receptor for advanced glycosylation end products (RAGE), Toll-like receptor (TLR)-2 and TLR-4 (Lee, Ku et al., 2012). The combination of HMGB1 with its particular receptor leads to the activation of nuclear factor-κB (NF-κB), which further upregulates the expression and release of inflammatory factors (78), thereby damage endothelial cells. Li et al. (79) showed that paeoniflorin significantly inhibited HMGB1 expression and release, down-regulated RAGE, TLR-2 and TLR-4 expression, and reduced LPC-induced NF-κB activity, which inhibited LPC-induced inflammatory factor production. It demonstrates that the mechanism behind this is primarily by suppressing the HMGB1-RAGE-/TLR-2-/TLR-4-NF-κB pathway, thereby suppressing endothelial cell damage and effectively interfered with the process of atherosclerosis.

In numerous studies over the past decade, TAK1 has been clearly demonstrated to play a key role in a range of physiological and biochemical processes (e.g., inflammation, regulation of immune system homeostasis and neurodevelopment) (80). Yu et al. (81) have demonstrated that paeoniflorin successfully inhibited TAK1 phosphorylation in TGFβ1 and IL-1β-stimulated endothelial cells, suppressed inflammatory factor expression by blocking the TAK1/NF-κB pathway and inhibited inflammatory factor expression.

#### 4.1.4 Paeoniflorin regulates endothelial cell-monocyte adhesion

Cell adhesion molecules (CAMs) are membrane receptor glycoproteins whose main function is to achieve cell adhesion on the endothelial surface (82). CAMs are composed of three main components: integrins, selectins and immunoglobulin superfamily. Among them, E-selectin, ICAM-1 and VCAM-1 are particularly important in the course of chronic inflammation and are consequently of major relevance in CVD (83). The mutual interaction between leukocytes and endothelial cells is a central event in the recruitment of leukocytes from blood vessels to undifferentiated tissues and can occur through three steps, namely scrolling, adhesion and migration. During this process, E-selectin is present on endothelial cells and is essential for leukocyte scrolling (84) and ICAM-1 is important for leukocyte adhesion and migration (85). Cell surface adhesion molecules (such as E-selectin and ICAM-1) are liberated during endothelial cell activation, and they are responsible for the recruitment of various leukocytes, exacerbating increased endothelial permeability and tissue damage (86). It is well known that selectin is a protein-binding molecule whose main function is to mediate the interaction between leukocytes and endothelial cells, creating strong adhesion and migration in endothelial cells. Endothelial cells strongly express E-selectin in response to stimulation by inflammatory factors (e.g., TNF-α and IL-1), which exacerbates adhesion (87). Simultaneously, Both ICAM-1 and VCAM-1 are transmembrane glycoproteins. ICAM-1 binds to integrins and promotes leukocyte migration toward and tight adhesion to endothelial cells, thereby affecting myosin contractility and p38 kinase activation (88). While VCAM-1, a member of the immunoglobulin superfamily, is expressed mainly in endothelial cells and is responsible for the recruitment of monocytes (89).

Chemokines are a category of minor cytokines or proteins responsible for leukocyte recruitment, adhesion, and activation. They are classified into the CC, CXC, CX3C, and XC subfamilies (90). Among chemokines, CCL2 (monocyte chemotactic protein-1, MCP-1) was the first human chemokine to be identified. MCP-1 and its receptor (CCR2) are essential for the recruitment of immune cells to reach the site of inflammation during the onset and development of the inflammatory response (91). Other well-studied chemokines include CCL5 (also called regulated upon activation, expressed, and secreted by normal T cells, RANTES), CCL20 (also called macrophage chemotactic protein 3α, MIP-3α), and CXCL8 (also called interleukin-8, IL-8), CXCL16 (also called scavenger receptor-binding phosphatidylserine and oxidized lipoprotein, SR-PSOX) and CX3CL1 (also called fractalkine), which can be released by activated endothelial cells to direct circulating leukocyte recruitment to the damaged site. Different signaling pathways assume different roles in the functioning of the above molecules. The most important of these is the MAPK signaling pathway. The MAPK family is composed of four members, including extracellular signal-regulated kinase (ERK), c-Jun N-terminal kinase (JNK), p38 and large map kinase 1 (BMK1)/ERK5 (92). Among them, those closely related to cell adhesion are mainly ERK1/2 and p38 (93). Another closely related activation pathway is the activation of NF-κB, which is present in the cytoplasm in an inactive form bound to members of the inhibitory κB (IκB) family. Stimulatory factors (e.g., LPS or TNF-α) leads to the phosphorylation and degradation of IκB, promote NF-κB translocation into the nucleus and its binding to target gene promoter regions, ultimately inducing the expression of cytokines and adhesion molecules (94, 95).

Chen et al. (96) found that paeoniflorin significantly blocked immune complex IC-induced expression of vascular endothelial E-selectin and ICAM-1, while for TNF-α-induced expression of adhesion molecules in human dermal microvascular endothelial cells (HDMEC), paeoniflorin showed some selectivity by inhibiting the activation of p38 and JNK thereby significantly inhibiting the expression of ICAM-1. The study by Jin et al. (97) also demonstrated that paeoniflorin downregulated the expression of ICAM-1 in TNF-α-stimulated HUVEC by inhibiting the activation of the NF-kB pathway, corroborating the anti-adhesive effect of paeoniflorin. Similarly, Wang et al. (98) investigated the function of paeoniflorin on oxidized LDL-induced adhesion molecule expression and showed that paeoniflorin significantly inhibited the molecular expression of E-selectin, VCAM-1 and ICAM-1 by enhancing autophagy and upregulating SIRT1 expression. Chen et al. (70) in an experiment investigating the response of paeoniflorin to endoplasmic reticulum stress found that paeoniflorin inhibited NF-κB activation, resulting in a significant inhibitory effect on MCP-1 effectively. Not coincidentally, Xu et al. (99) found that paeoniflorin significantly down-regulated E-selectin expression at the gene and protein levels. Paeoniflorin also inhibited the phosphorylation of JNK and p38 in a dose dependent manner, thereby suppressed the MAPK signaling pathway. In addition, paeoniflorin significantly inhibited the phosphorylation and degradation of IκB-α, which in turn also inhibited the activation of NF-κB and reduced the expression of ICAM-1. Paeoniflorin significantly reduced the adhesion of human acute monocytic leukemia cells (THP-1) or human acute promyelocytic leukemia cells (HL-60) to LPS-stimulated HUVEC. In another study, Chen et al. conducted a detailed study on TNF-α-induced mRNA expression of chemokines in a human microvascular endothelial cell (HMEC-1), and they found that paeoniflorin significantly inhibited CCL2, CCL5, CCL20, CXCL8, CXCL16 and CX3CL1 mRNA expression, reduced chemokine secretion, and successfully inhibited leukocyte migration. Also, their study revealed that only high doses of paeoniflorin inhibited the release of chemokines (e.g., CCL5, CCL20, CXCL16, and CX3CL1), and these data suggest that the inhibitory effect of paeoniflorin on chemokines is stronger at the transcriptional level than at the translational level and post-translational secretion process.

#### 4.1.5 Paeoniflorin regulates the survival and fate of endothelial cells

Previous studies have found that paeoniflorin can affect the fate of endothelial cells through apoptosis and autophagy. Autophagy is a double-edged sword in that its activation tends to increase VEC survival under stressful conditions (100). However, if autophagy is excessive, it can lead to endothelial cell damage and death (101). The autophagic process involves many genes, one of the key ones is Microtubule-associated protein light chain 3 (LC3), which is important for autophagosome formation (102). When autophagy is started, the cytoplasmic form of LC3-I is converted into the membrane-associated form of LC3-II, which is the major protein of autophagy (103).The role of the p62 protein is to attach LC3 proteins to ubiquitinated substrates, thereby incorporating them into the intact phagosome through autophagic lysosomal degradation (104). As another inseparable concept of cell fate, the process of apoptosis, also called programmed cell death, involves a complex series of signaling cascade responses that culminate in an orderly termination of the cell mediated by signals. Although apoptosis ends in cell death, it is actually critical for maintaining the continued health of the entire organism (105), and when apoptosis is deregulated, it can lead to atherosclerosis (106). In atherosclerotic patients, apoptosis of endothelial cells leads to a series of adverse consequences, such as plaque erosion, instability and thrombosis, that further lead to the development of acute symptoms (107). In the above process, the MAPK cascade mediates the activation of several transcription factors, such as NF-κB, and modulates the performance of apoptosis-related proteins, such as caspase-3 (that induces apoptosis) and B-cell lymphoma-2 (Bcl-2) (that prevents apoptosis) (108). Ji et al. (109) showed that paeoniflorin prevented CoCl_2_-induced hypoxia-inducible factor 1α (HIF-1α) accumulation and downregulated p53 and BNIP3 expression in endothelial cells, reducing apoptosis and improving endothelial cell viability in a dose-dependent manner. Because HIF-1α, the active subunit of HIF-1, can inhibit apoptosis by activating a variety of anti-apoptotic genes, it is associated with the development of human atherosclerosis (110).

Two other pathways of action that should not be neglected are SIRT1 and RAGE. Endothelial-specific SIRT1 is a bona fide anti-atherosclerotic factor that effectively improves endothelial cell survival and function (111). A study by Wang et al. (98) found that paeoniflorin attenuated oxidized low-density lipoprotein-induced endothelial cell apoptosis by upregulating enhanced autophagy of SIRT1 in HUVECs. Because the accumulation of advanced glycosylation end products (AGEs) can trigger oxidative stress, oxidized lipids, inflammation, metabolic stress states and apoptosis, leading to endothelial cell dysfunction. This dysfunction, accompanied by vascular damage, can lead to increased atherosclerotic plaque formation (112). RAGE is a positive regulator of autophagy during oxidative stress and a negative regulator of apoptosis (113). Chen et al. (114) indicated that pretreatment with paeoniflorin increased LC3-II and RAGE levels but decreased p62 expression. Paeoniflorin protected HUVEC from AGE-modified bovine serum albumin (AGE-BSA)-induced damage by upregulating autophagy and promoting the completion of autophagic flux. RAGE played important role in this autophagic protection. These research efforts suggest that paeoniflorin may be a new therapeutic tool for patients suffering from atherosclerosis and other systemic diseases, such as diabetes mellitus.

CypD also has a protective effect against apoptosis (115). Ou et al. (69) investigated apoptosis in endothelial cells induced by H_2_O_2_ and found that paeoniflorin significantly decreased the expression of caspase-3 and Bax while increased the expression of CypD and Bcl2 expression, which successfully prevented apoptosis. Jiang et al. (64) showed that paeoniflorin protected HUVEC from apoptosis by stimulating the nuclear factor erythroid 2-related factor 2 (Nrf2)/heme oxygenase 1 (HO-1) pathway, with the possible mechanism mainly promoting the expression of Bcl-2 and inhibiting the expression of Bax and cleavage of caspase-3.

#### 4.1.6 Paeoniflorin blocks endothelial-mesenchymal transition (EndMT) in endothelial cells

Current studies generally agree that endothelial-mesenchymal transition (EndMT) is one of the critical mechanisms leading to endothelial cell dysfunction (116). Endothelial growth is characterized by two aspects for metrics, on the one hand the acquisition of mesenchymal cell markers (e.g., α-SMA, Vimentin and N-cadherin) and on the other hand the loss of endothelial cell markers (e.g., CD31, VE-cadherin and eNOS) (117). Recently, researchers have shown that EndMT promotes the development of atherosclerosis (118). Yu et al. (81) showed that prophylactic paeoniflorin administration restored monocrotaline (MCT)-induced downregulation of VE-cadherin and eNOS while inhibited MCT-induced upregulation of α-SMA and vimentin, thereby blocked EndMT and potently counteracting the development of the atherosclerotic process.

### 4.2 Paeoniflorin and smooth muscle cell function

**Figure 6:**
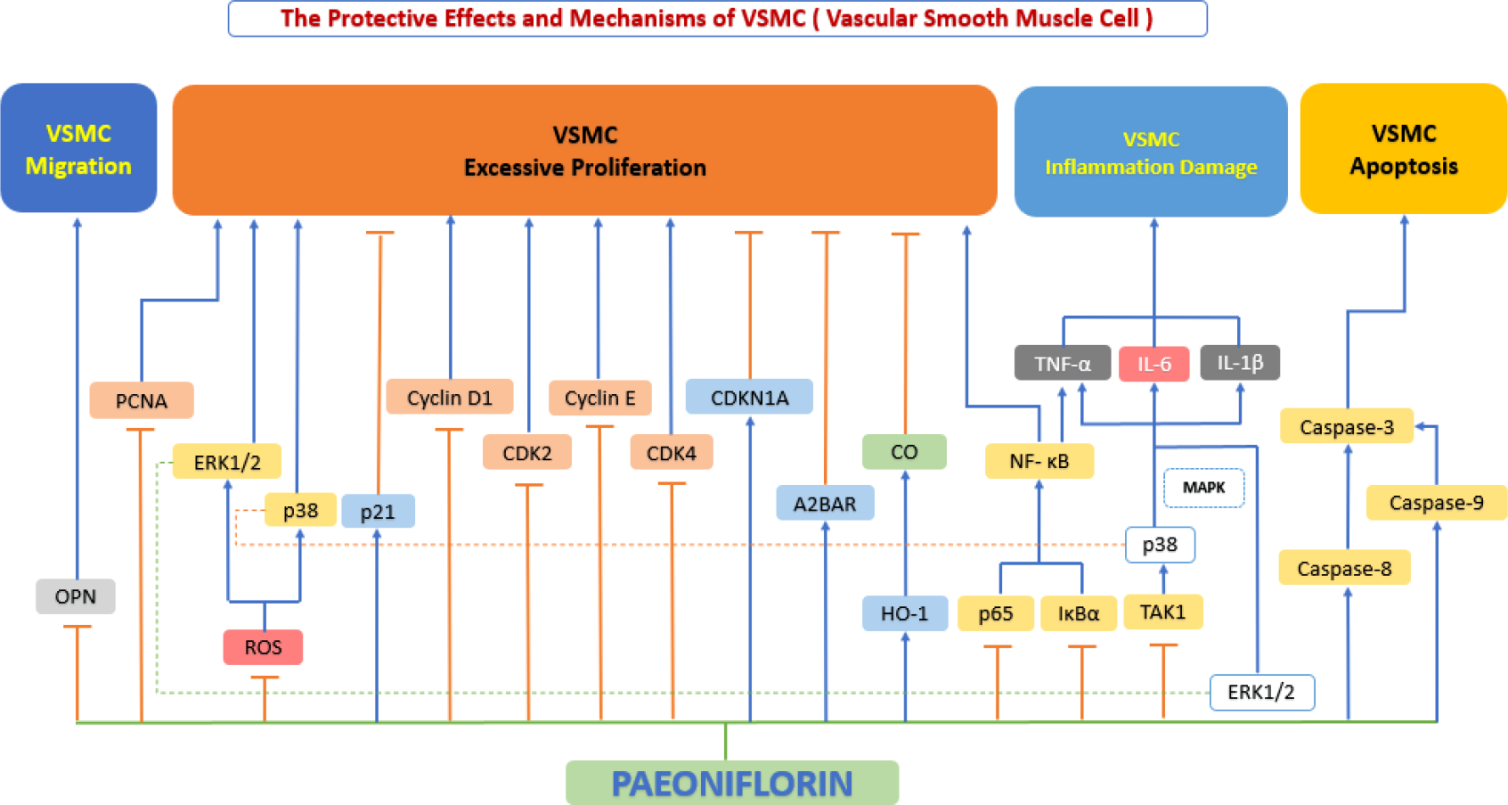
The protective effects and mechanisms of paeoniflorin on smooth muscle cells. Paeoniflorin prevents vascular smooth muscle cell (VSMC) dysfunction by inhibiting excessive VSMC migration and excessive proliferation, suppressing inflammatory factor secretion, and regulating VSMC survival, this in turn exerts an anti-atherosclerotic effect. CDK2, cyclin-dependent kinase 2; CDK4, cyclin-dependent kinase 4; CDKN1A, cyclin-dependent kinase inhibitor 1A; CO, carbonic oxide; ERK1/2, extracellular signal-regulated kinase 1/2; HO-1, Heme Oxygenase 1; IL-1β, interleukin 1β; IL-6, interleukin 6; IκBα, inhibitor of kappa B alpha; MAPK, mitogen-activated protein kinase; NF-κB, the nuclear factor-kB; OPN, osteopontin; p38, p38 mitogen-activated protein kinase (MAPK); PCNA, proliferating cell nuclear antigen; ROS, reactive oxygen species; TAK1, transforming growth factor beta-activated kinase1; TNF-α, tumor necrosis factor α.

In the pathological development of atherosclerosis, abnormal proliferation, migration and phenotypic conversion of vascular smooth muscle cells (VSMC) are prominent manifestations of VSMC dysfunction and are closely associated with the formation of neointima and the development of atherosclerosis (119). An increasing stream of studies has shown that paeoniflorin can prevent VSMC dysfunction by inhibiting excessive VSMC migration and excessive proliferation, suppressing inflammatory factor secretion, and regulating VSMC survival (**Figure 6**).

#### 4.2.1 Paeoniflorin inhibits smooth muscle cell proliferation and migration

The follow-up activity after endothelial cell injury is abnormal VSMC proliferation and migration (120). Along with the above process, a large amount of cytokines/chemokines/growth factors are released to compensate and repair the corresponding endothelial damage, including VEGF, basic fibroblast growth factor (bFGF), transforming growth factor-β (TGF-β) and platelet-derived growth factor (PDGF) (119). By binding to VSMC membrane receptors, these growth factors trigger phosphorylation of receptors downstream of the VEGF or PDGF-B pathways (121), thereby evoking activation of several downstream signaling pathways, such as the MAPK signaling pathway (122). In VSMC, activation of MAPK signaling pathways, such as the ERK1/2 pathway and the p38 MAPK pathway, can lead to cell proliferation and migration and can change from a contractile to a proliferative phenotype (123). Yu et al. (81) also studied PDGF-BB-induced VSMC proliferation and analyzed ERK1/2, p38 MAPK, and p65 NF-κB pathways in detail and found that paeoniflorin inhibited the phosphorylation of the above pathways in a dose-dependent manner and suppressed PDGF-BB-induced proliferation and migration of PASMC. The experiments of Guo et al. also demonstrated the aforementioned effect of paeoniflorin (124). Another study worth noting was that, Li et al. (125) investigated the effects of paeoniflorin on ox-LDL-induced VSMC proliferation and migration and showed that paeoniflorin blocked p38, ERK1/2, and NF-κB phosphorylation (without inhibiting JNK phosphorylation) and blocked the cell cycle in S phase, upregulated HO-1 expression, decreased proliferating cell nuclear antigen (PCNA) expression and antagonized oxidized LDL-induced VSMC proliferation and migration.

Our literature review found that OPN and adenosine receptors also play an important role. OPN is a marker gene for the conversion of vascular smooth muscle cells from a constrictive to a proliferative phenotype, and the expression of OPN is strongly correlated with the migratory capacity of VSMC (126). The development of the above processes leads to VSMC proliferation and thickening of the endothelium (127), further deepening the development of atherosclerosis. Adenosine receptors are extracellular G protein-coupled receptors (classes include A1, A2A, A2B and A3), and primarily mediate the effects of adenosine (128). A2B adenosine receptor (A2BAR) has a low binding affinity compared to other subtypes (129). A study showed that hypoxia down-regulated the A2AAR (high-affinity) and up-regulated the A2BAR (low-affinity) in HUVECs (130). Adenosine signaling *via* A2BAR has been proven to inhibit SMC proliferation (131). Qian et al. (132) reported that paeoniflorin successfully inhibited the proliferation of pulmonary artery smooth muscle cells by activating A2BAR and inducing its expression. Fan et al. (133) showed that paeoniflorin blocked the cell cycle progression of VSMCs from G0/G1 to S phase, downregulated PDGF-BB-induced CDK4, CDK2, cell cycle protein D1, cytokinin E, and cyclin-dependent kinase inhibitor 1A (CDKN1A) in a dose- and time-dependent way, as well as PDGF-BB-induced VSMC proliferation. At the same time, paeoniflorin significantly suppressed cell migration by downregulating OPN gene expression, and revealed that the mechanism behind this may be related to the fact that paeoniflorin significantly decreased the level of phosphorylation of p38 and ERK1/2 in a concentration-dependent manner, but not the phosphorylation of JNK.

#### 4.2.2 Paeoniflorin inhibits the secretion of inflammatory factors in smooth muscle cells and regulates the cell fate

VSMC are pivotal players in early and late atherosclerosis (134). Recent studies in genetics suggest that VSMC-derived macrophage-like cells may promote inflammation (135). The main pro-inflammatory cytokines (such as IL-1β, TNF-α, and IL-6) more closely associated with the developmental process of atherosclerosis (136). Activation of the NF-κB pathway will lead to overproduction of the three aforementioned pro-inflammatory cytokines (137). Yu et al. (81) showed that paeoniflorin successfully inhibited the phosphorylation of TAK1 in PDGF-BB-stimulated human pulmonary artery smooth muscle cells (PASMC) and suppressed inflammatory factor expression by blocking the TAK1-MAPK/NF-κB pathway. Li et al. (125) investigated the effect of paeoniflorin on ox-LDL-induced inflammatory cytokines in VSMC and showed that paeoniflorin dose-dependently blocked p38, ERK1/2, and p65 NF-κB phosphorylation and effectively blocked IL-6 and TNF-αexpression, with the strongest inhibition of IL-6.

VSMC apoptosis is induced by pro-inflammatory cytokines, oxidized LDL, high levels of NO, and mechanical damage. The inefficient clearance of apoptotic VSMC leads to secondary necrosis and subsequent inflammation, triggering plaque rupture and adverse consequences (138). In addition, excessive apoptosis of VSMC cells leads to calcification and inflammation, thereby, promotes atherosclerosis (139). Cysteine aspartate-specific protease (Caspase)-8 has long been recognized as the promoter of apoptosis (140), and caspase-9 plays a critical role in the apoptosis pathway of endogenous origin (141). Guo et al. (124) demonstrated that paeoniflorin was able to induce VSMC apoptosis by significantly enhancing the expression of caspase-3/-8/-9 in a concentration-dependent manner.

### 4.3 Paeoniflorin and monocyte function

**Figure 7:**
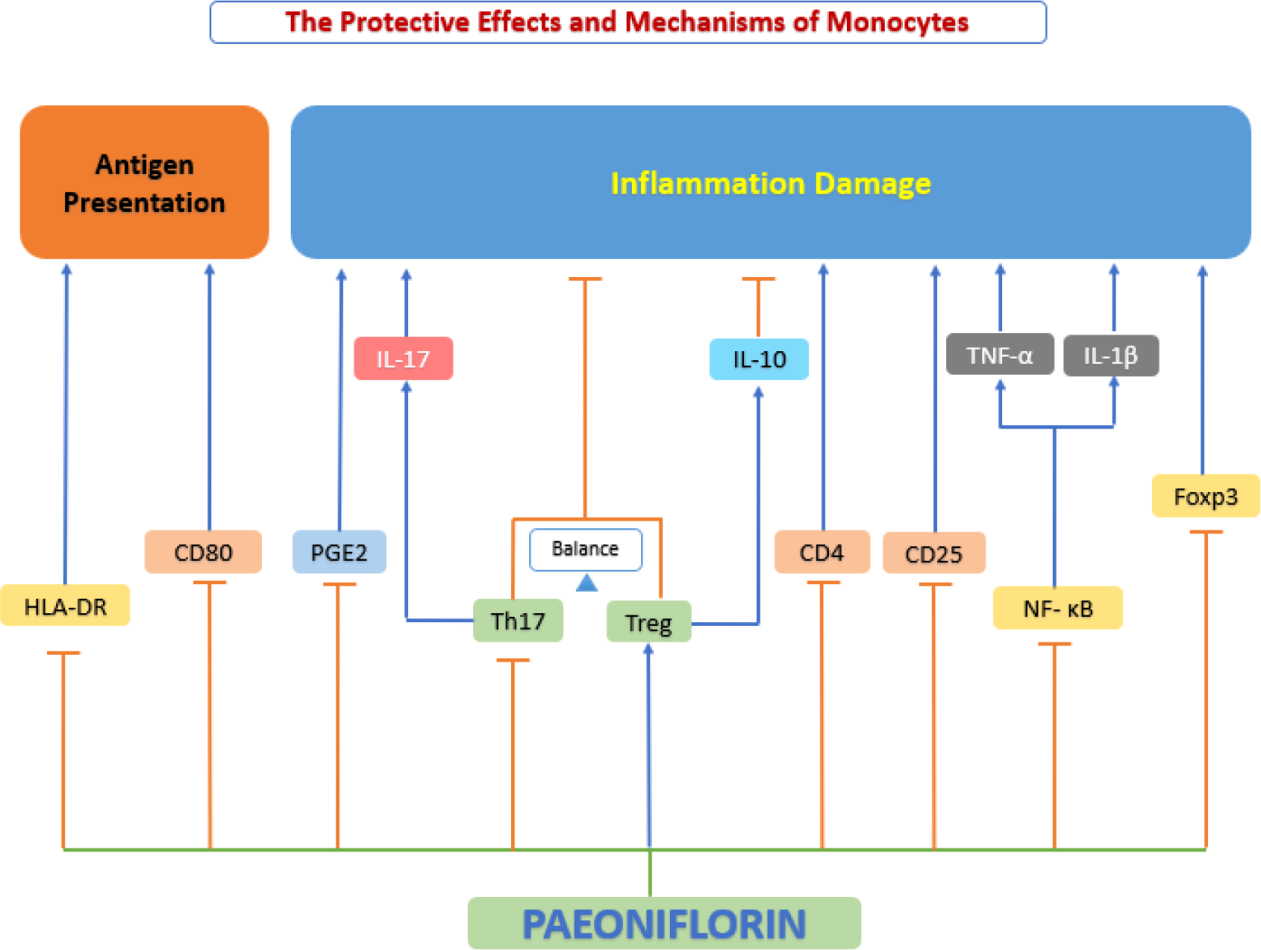
The protective effects and mechanisms of paeoniflorin on monocyte. Paeoniflorin affects the progression of atherosclerosis by inhibiting phagocytosis and antigen presentation, over proliferation and inflammatory factor secretion by monocytes. Foxp3, forkhead box P3; HLA-DR, human leukocyte antigen DR; IL-10, interleukin 10; IL-17, interleukin 17; NF-κB, the nuclear factor-kB; PGE2, prostaglandin E2; Th17, T helper type 17 cell; Treg, Regulatory T cells.

Atherosclerotic lesions are closely associated with the progression of continuous enrollment of circulating vascular monocytes, which are differentiated into macrophages inside the atherosclerotic plaque (142). In recent years, an increasing number of studies have shown that paeoniflorin affects the progression of atherosclerosis by inhibiting phagocytosis and antigen presentation, over proliferation and inflammatory factor secretion by monocytes (**Figure 7**).

#### 4.3.1 Paeoniflorin inhibits phagocytosis and antigen presentation by monocytes

Phagocytosis is an evolutionarily conserved process that satisfies feeding mechanisms in protozoa. However, in postnatal animals, phagocytosis diversifies to perform multiple organismal roles, including immune defense, tissue homeostasis, and remodeling (143). Microbial fragments remaining after phagocytosis and digestion can act as antigens that can be integrated into Major histocompatibility complex (MHC) molecules and then transferred to the cell surface of monocytes. This is an important process known as antigen presentation, which is important for activation Adaptive immune responses against viruses are critical (144). It has been shown that antigen-presenting ability is closely related to the expression of human Leukocyte class II DR antigens (HLA-DR) and CD80 (145). Wang et al. (146) revealed that paeoniflorin inhibited the activation of rhIL-1β-stimulated human peripheral blood monocytes, suppressed monocyte phagocytosis, and downregulated the expression of HLA-DR and CD80, inhibited the antigen-presenting ability of monocytes.

#### 4.3.2 Paeoniflorin inhibits monocyte proliferation and inflammatory factor secretion

The proliferation of monocytes provides abundant raw material for the subsequent evolution into macrophages in the course of atherosclerosis (147). In addition to this, the balance of two important Th cell subtypes, Th17 and regulatory T cells (Treg) plays a critical role in many inflammatory illness, with the Th17 phenotype associated with inflammation and the Treg phenotype counteracting it to some degree (148). IL-17 is an important cytokine produced by Th17 cells (149), while Treg can produce IL-10 (150), and they synergistically regulate inflammatory responses. In addition, it has been shown that activation of phagocytosis leads to the production of typical pro-inflammatory cytokines (e.g., TNF-α, IL-1β) by monocytes (151). Dai et al. (152) evaluated the proliferative response and anti-inflammatory capacity of paeoniflorin on interleukin (IL)-1β protein (rhIL-1β) -induced peripheral blood mononuclear cells (PBMC) and showed that paeoniflorin could significantly inhibit rhIL-1β-stimulated PBMC proliferation levels, down-regulate IL-17 production, and up-regulate IL-10 production *in vitro*. These results suggest that paeoniflorin can exert anti-inflammatory effects by inhibiting abnormal proliferation and regulating the balance of Th17 with regulatory T cells (Treg). In addition, Wang et al. (146) also showed that paeoniflorin may inhibit rhIL-1β stimulated production of Prostaglandin E2 (PGE2) and TNF-α, reducing chronic inflammation by inhibiting human monocytes.

### 4.4 Paeoniflorin and macrophage function

**Figure 8:**
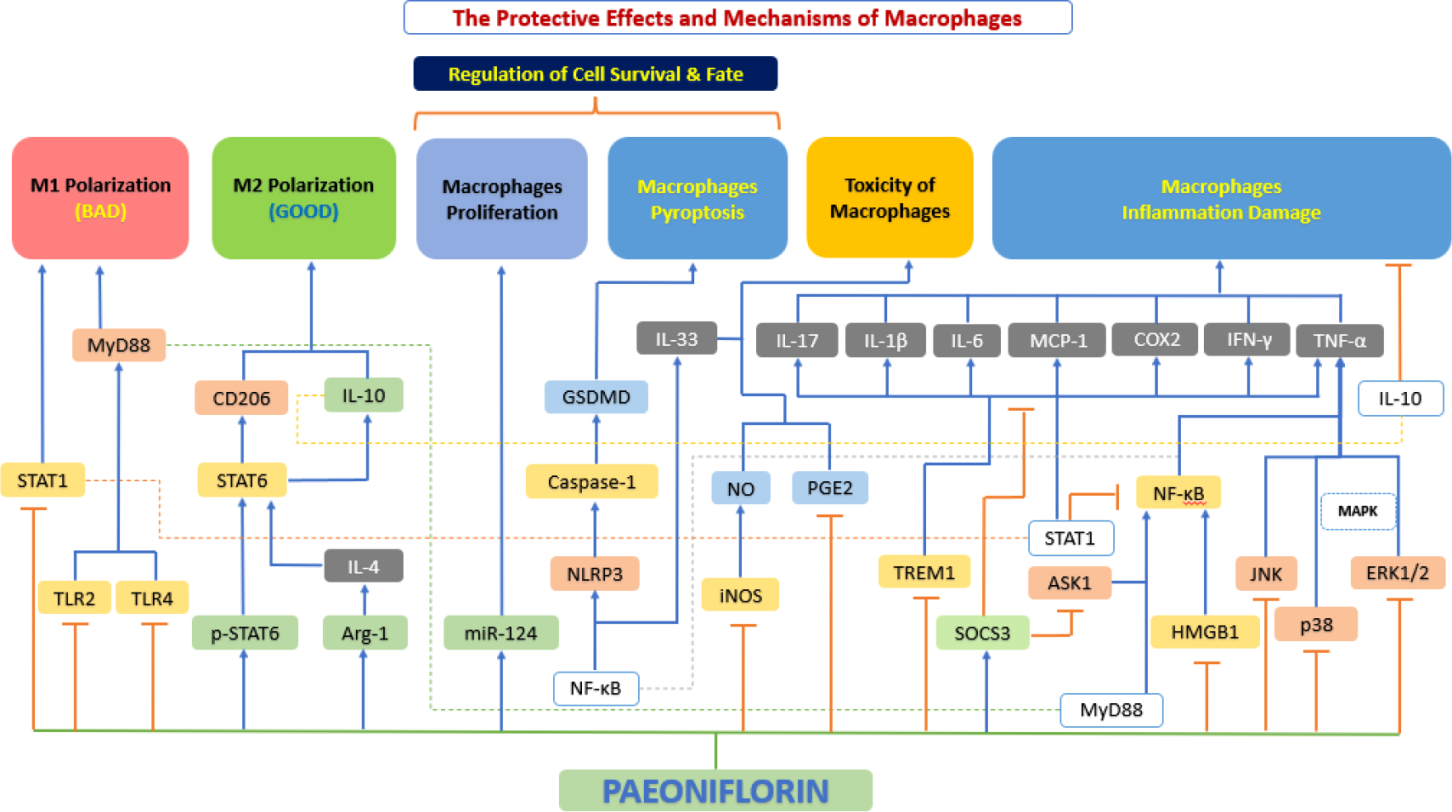
The protective effects and mechanisms of paeoniflorin on macrophages. Paeoniflorin has a protective effect on macrophages, which is achieved by regulating the phenotypic transformation of macrophages, inhibiting macrophage inflammatory factor secretion, regulating macrophage survival and fate, inhibiting macrophage proliferation and blocking foam cell formation. Arg-1, arginase 1; ASK1, apoptosis signal-regulating kinase 1; COX2, cyclooxygenase-2; ERK1/2, extracellular signal-regulated kinase 1/2; GSDMD, gasdermin D; HMGB1, high mobility group box 1; IFN-γ, interferon-γ; IL-10, interleukin 10; IL-10, interleukin 10; IL-1β, interleukin 1β; IL-33, interleukin 33; IL-4, interleukin 4; IL-6, interleukin 6; iNOS, inducible nitric oxide synthase; JNK, c-Jun N-terminal kinase; MAPK, mitogen-activated protein kinase; MCP-1, monocyte chemoattractant protein-1; miR-124, microRNA-124; MyD88, myeloid differentiation primary response 88; NF-κB, the nuclear factor-kB; NLRP3, NOD-like receptor 3; NO, nitrous oxide; p38, p38 mitogen-activated protein kinase (MAPK); PGE2, prostaglandin E2; p-STAT6, phosphorylation signal transducer and activator of transcription 6; SOCS3, suppressor of cytokine signaling 3; STAT1, signal transducer and activator of transcription 1; STAT1, signal transducer and activator of transcription 1; STAT6, signal transducer and activator of transcription 6; TLR2, toll-like receptor 2; TLR4: toll-like receptor 4; TNF-α, tumor necrosis factor α; TREM1, triggering receptor expressed on myeloid 1.

Macrophages play an important role in all stages of atherosclerosis, from the initiation and expansion of the lesion, through the necrosis and clinical manifestations of atherosclerosis leading to rupture, to the regression of the atherosclerotic pathologies (153). Recent and growing research evidence suggests that paeoniflorin has a protective effect on macrophages, which is achieved by regulating the phenotypic transformation of macrophages, inhibiting macrophage inflammatory factor secretion, regulating macrophage survival and fate, inhibiting macrophage proliferation and blocking foam cell formation (**Figure 8**).

#### 4.4.1 Paeoniflorin regulates the phenotypic transformation of macrophages

Macrophages are usually divided into two atherosclerosis-related categories: inflammatory macrophages (M1) and healing macrophages (M2) (154). Interferon γ (IFN-γ) or LPS can stimulate M0 macrophages to produce an M1 phenotype to secrete pro-inflammatory cytokines and ROS, or an M2 phenotype induced by interleukin-4 (IL-4)/IL-13 to express anti-inflammatory cytokines and tissue repair (155). Notably, polarized macrophages have been found to reverse in adapting to changes in the microenvironment (156). Activated STAT1, a pivotal transcription factor in the JAK-STAT pathway, primarily mediates M1 polarization (157), while STAT6 activation is involved in M2 polarization (158). Wang et al. (159) showed that paeoniflorin inhibits STAT1 phosphorylation to reduce M1 polarization and activates STAT6 through elevated p-STAT6 to participate in M2 polarization and regulate macrophage phenotype through STAT signaling. Studies by Zhai et al. (160) and Shao et al. (161, 162) have also demonstrated the same conclusion.

#### 4.4.2 Paeoniflorin inhibits macrophage inflammatory factor secretion

Closely linked to the aforementioned polarization of macrophages, polarized macrophages promote the secretion of various inflammatory factors, thereby exacerbating the response in the process of atherosclerosis (163). Cao et al. (164) demonstrated that paeoniflorin significantly attenuated the phosphorylation of NF-κB signaling pathway-related factors (e.g., p65 and I-κBα), thereby reducing the expression of TNF-α, IL-1β, and IL-6 in macrophages. The same results were shown by Fang et al. (165). Zhang et al. (166) experimentally confirmed that paeoniflorin not only reduced the production of TNF-α and IL-6 by downregulating TLR4 expression and inhibiting NF-κB and MAPK signaling pathways but also synchronously downregulated MCP-1, Cox2, IFN-γ, TNF-α, IL-6 and IL-17 expression and overall inhibit inflammation. Studies by Wang et al. (167), Shao et al. (161) and Zhang et al. (168) also corroborate this conclusion. Liu et al. (169) further investigated the cause of negative feedback regulation of LPS-induced MAPK in macrophages by paeoniflorin and found that paeoniflorin achieved inhibition of LPS-induced macrophage inflammation by increasing the expression of suppressors of cytokine signaling 3 (SOCS3), a common inhibitory signaling regulatory protein. Sheng et al. (170) also investigated the effect of paeoniflorin on SOCS3 in C57BL/6 mice, demonstrating the significance of SOCS3 as an “inflammatory braking” factor. Jiang et al. (171) demonstrated that paeoniflorin can downregulate the levels of TNF-α, IL-6, and High mobility group box-1 protein (HMGB1, formerly known as HMG-1) in macrophages by inhibiting the IκB kinase pathway and modulating the NF-κB signaling pathway in a dose dependent manner. It also upregulated serum levels of the anti-inflammatory factor IL-10, which effectively counteracted inflammation. The same conclusion was reached by Wang et al. (159) in their experiment.

#### 4.4.3 Paeoniflorin regulates the survival and fate of macrophages

The regulation of macrophage survival and fate by paeoniflorin mainly covers three aspects: proliferation, apoptosis, and pyroptosis. Macrophage apoptosis is a non-negligible feature of atherosclerotic plaque progression, especially in early stages of the lesion, and macrophage apoptosis greatly inhibits the progression of atherosclerotic plaque (172). It has been shown that microRNA (miRNA)-124 is a key regulator of inflammation and immunity (173). It has been shown that miR-124 can effectively inhibit the proliferation of macrophages (174), and is essential for the inducibility and maintenance of the M2 phenotype of macrophages (175). A study by Huang et al. (176) found that paeoniflorin inhibited proliferation and inflammation of THP-1 cells and promoted their apoptosis through upregulation of miR-124, thereby inhibited atherosclerotic plaque progression.

Pyroptosis is an inflammatory form of programmed cell death activated by the caspase family (e.g., caspase-1) and accompanied by cell swelling, osmotic lysis, and activation of NOD-like receptor family pyrin domain containing 3 (NLRP3) inflammatory vesicles (177). Excessive pyroptosis causes the release of large amounts of inflammatory cytokines (178). By reducing the pyroptosis of cells or tissues, it is beneficial to reduce the local inflammatory response of organisms and promote the repair of damaged tissues and disease recovery (179). Xu et al. (180) focused on macrophage pyroptosis, and their study found that paeoniflorin monomer derivative (MDP) could inhibit macrophage polarization and pyroptosis, and the mechanism behind this may be related to TLR4/NLRP3/GSDMD signaling pathway inhibition.

#### 4.4.4 Paeoniflorin inhibits the migration and aggregation of macrophages, and blocks macrophage foaminess

Macrophages play a central role in the development of atherosclerotic cardiovascular disease (ASCVD), including coronary artery disease, peripheral artery disease, cerebrovascular disease and aortic atherosclerosis. In each vascular bed, migration and aggregation of macrophages help to maintain the inflammatory local reaction, spread plaque development and facilities thrombosis (181). Zhang et al. (182) showed that paeoniflorin significantly inhibited macrophage migration. Shao et al. also experimentally confirmed the anti-macrophage migration ability of paeoniflorin (161). While Zhang et al. experimentally demonstrated that paeoniflorin could significantly reduce macrophage aggregation (168).

Foam cell formation and accumulation are important markers of early atherosclerotic lesions, and previous studies have shown that their formation within the intima is mainly due to impaired cholesterol efflux or excessive uptake of ox-LDL by macrophages (183). The conversion of macrophages to foam cells is consistently accompanied by an overproduction of pro-inflammatory cytokines and chemokines (4), which deepens and aggravates atherosclerosis. Li et al. (125) showed that paeoniflorin successfully blocked oxLDL-induced foam cell formation in macrophages.

#### 4.4.5 Other protective effects of paeoniflorin on macrophages

In addition to the various mechanisms mentioned above for macrophages, paeoniflorin has some other protective effects, mainly including both blunting the immune response to atherosclerosis and repairing damaged cellular DNA. IL-33 is a newly discovered member of the IL-1 family, first identified in 2005 (184). IL-33 is usually released after cellular damage and acts as an “alarm” to early warning the immune system and activate the repair process (185). The inhibition of IL-33 production can blunt the immune response characteristic of atherosclerosis, thus exerting a cytoprotective effect (186). Li et al. (187) found that paeoniflorin could reduce IL-33 in two ways. Firstly, paeoniflorin suppressed IL-33 production by reducing TLR4 expression and inhibiting LPS-induced NF-κB activation and P38MAPK phosphorylation. In addition, paeoniflorin further reduced IL-33 production by inhibiting Ca^2+^ influx and triggering subsequent inhibition of NF-κB and P38MAPK activation. Kim et al. (188) then investigated the issue of LPS-induced cytotoxicity and found that paeoniflorin protected macrophages from LPS-induced toxicity by increasing macrophage viability, reducing NO and PGE2, and repairing macrophage DNA in a dose-dependent way.

### 4.5 Paeoniflorin and platelet function

**Figure 9:**
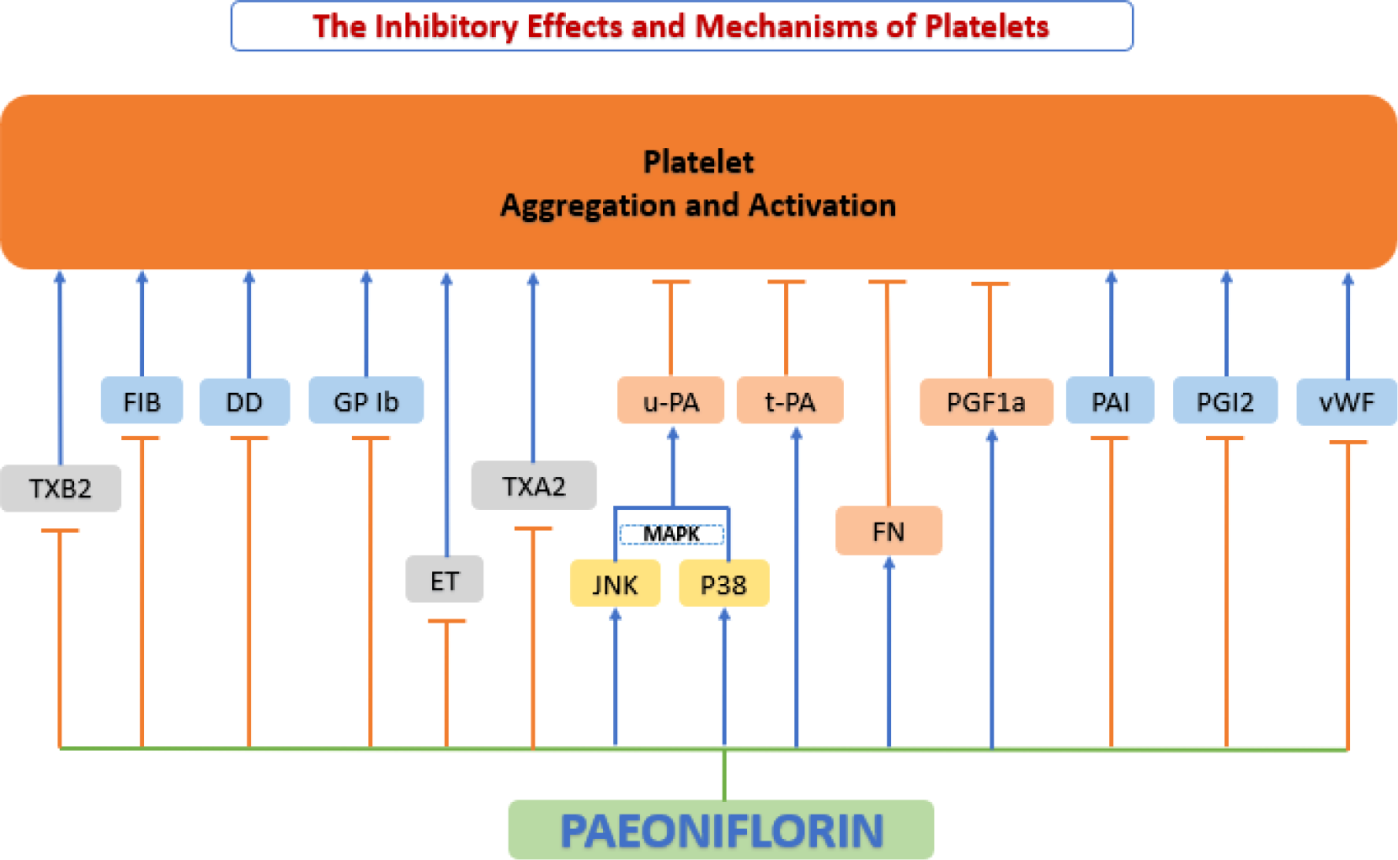
Anti-platelet function of paeoniflorin. Paeoniflorin exerts an important antithrombotic function by inhibiting platelet aggregation and activation. DD, d-dimer; ET, endothelin; FIB, fibrinogen; FN, fibronectin; GP Ib, glycoprotein Ib; JNK, c-Jun N-terminal kinase; MAPK, mitogen-activated protein kinase; p38, p38 mitogen-activated protein kinase (MAPK); PAI, plasminogen activator inhibitor; PGF1a, 6-keto prostaglandin Fla; PGI2, prostacyclin; t-PA, tissue-type plasminogen activator; TXA2, thromboxane A2; TXB2, thromboxane B2; u-PA, urokinase-type plasminogen activator; vWF, von Willebrand factor.

When some environmental factors change (e.g., alteration of blood rheological parameters, generation of blood turbulence, upregulation of procoagulant substances in atherosclerotic lesions, etc.), platelets are activated, trigger a coagulation cascade effect that leads to the formation of arterial thrombi. These thrombi may lead to vascular obstruction and eventually to life-threatening acute coronary events (189). In contrast to platelet activation pathways that are dependent on physiological agonists, shear stress-induced platelet aggregation (SIPA) occurs only in pathological conditions (e.g., vascular stenosis or atherosclerotic lesions) (190). Under high shear stress conditions, binding of vWF to glycoprotein (GP) Ib leads to signal transduction, which activates platelets (191). When platelets are activated, degranulation of the platelet surface and GP IIb/IIIa activation leads to the stabilization of platelet aggregation and subsequent acceleration of thrombosis (192, 193). In clinical practice, prothrombin time (PT), activated partial thromboplastin time (aPTT), thrombin time (TT), and fibrinogen (FIB) are the commonly used anticoagulant-related measures (194). Recent studies have shown that paeoniflorin exerts an important antithrombotic function by inhibiting platelet aggregation and activation (**Figure 9**).

Ngo et al. (195) showed that paeoniflorin inhibited SIPA through the interaction of vWF with platelet surface GP Ib, and selectively inhibited shear stress-induced platelet aggregation and activation and significantly prevented thrombosis with no increased risk of hemorrhage. Ye et al. (196) based on experiments in spontaneously hypertensive rats (SHR) also found that paeoniflorin treatment significantly upregulated 6-keto-prostaglandin F1a (6-keto-PGF1a), fibronectin (FN), and urokinase-type plasminogen activator (uPA) levels. Paeoniflorin treatment also inhibited the expression of fibrinogen (FIB), D-dimer (DD) and thromboxane B2(TXB2), thereby suppressed platelet aggregation and activation, where uPA may be mediated through the regulation of p38 and JNK MAPK signaling pathways. Another study based on Wistar rats also showed that paeoniflorin inhibited platelet aggregation and activation by increasing tissue-type plasminogen activator (tPA) activity and decreasing the production of plasminogen activator inhibitor (PAI), prostaglandin I2 (PGI2), and thromboxane A2(TXA2) (197).

Zhu et al.’s experiments based on New Zealand white rabbits (198) and Xie et al.’s experiments based on Sprague-Dawley (SD) rats (199) focused on the platelet anticoagulation of paeoniflorin, and their experimental results showed that paeoniflorin significantly prolonged TT, APTT and PT and reduced FIB levels *in vivo*, demonstrating a good antithrombotic effect. This conclusion was also confirmed by Fang et al. based on Swiss mice (165). In addition, Xie’s study also showed that paeoniflorin significantly lower the levels of ET while significantly elevate eNOS content, and the activation of eNOS promoted the sustained release of NO, resulting in endothelium-dependent vasodilation (199). Therefore, paeoniflorin can be considered a safe, thrombosis-reducing vasoprotective agent.

### 4.6 Other protective effects and mechanisms

#### 4.6.1 Homeostatic regulation of mast cells

Mast cells have been considered primarily as effectors of allergy. In the last two decades, the role of mast cells in other processes (physiological/pathological) has been gradually acknowledged. Mast cells interact with the surrounding microenvironment, and release a series of bioactive mediators to specifically recognize and respond to various stimuli (200). Recently, a large volume of evidence has demonstrated that mast cells have a significant impact on cardiovascular disease, particularly on the development and progression of atherosclerotic plaques (201). When activated, mast cells release a series of substances, such as growth factors, histamine and chemokines, which cause matrix degeneration, apoptosis, and inflammatory cell recruitment, ultimately leading to a series of adverse cardiovascular events (202).

IgE (FcRI) on the surface of mast cells can cross-link with high-affinity receptors for immunoglobulin E (IgE) to secrete granules that are filled with inflammatory mediators and can induce a variety of biological activities (203). A study by Zhang et al. (204) found that paeoniflorin inhibited *in vitro* DNP-BSA-induced mast cell degranulation, and inhibited IgE-mediated histamine release and calcium inward flow. Paeoniflorin also downregulates the phosphorylation levels of key kinases such as ERK1/2, P38 and Phospholipase C (PLC), and reduces IgE-mediated release 5-hydroxytryptamine (5-HT; serotonin), MCP-1, IL-8, and TNF-α, suppressed the local inflammatory response. Meanwhile, they found through molecular docking experiments that paeoniflorin may be a potential FcRI ligand.

**Figure 10:**
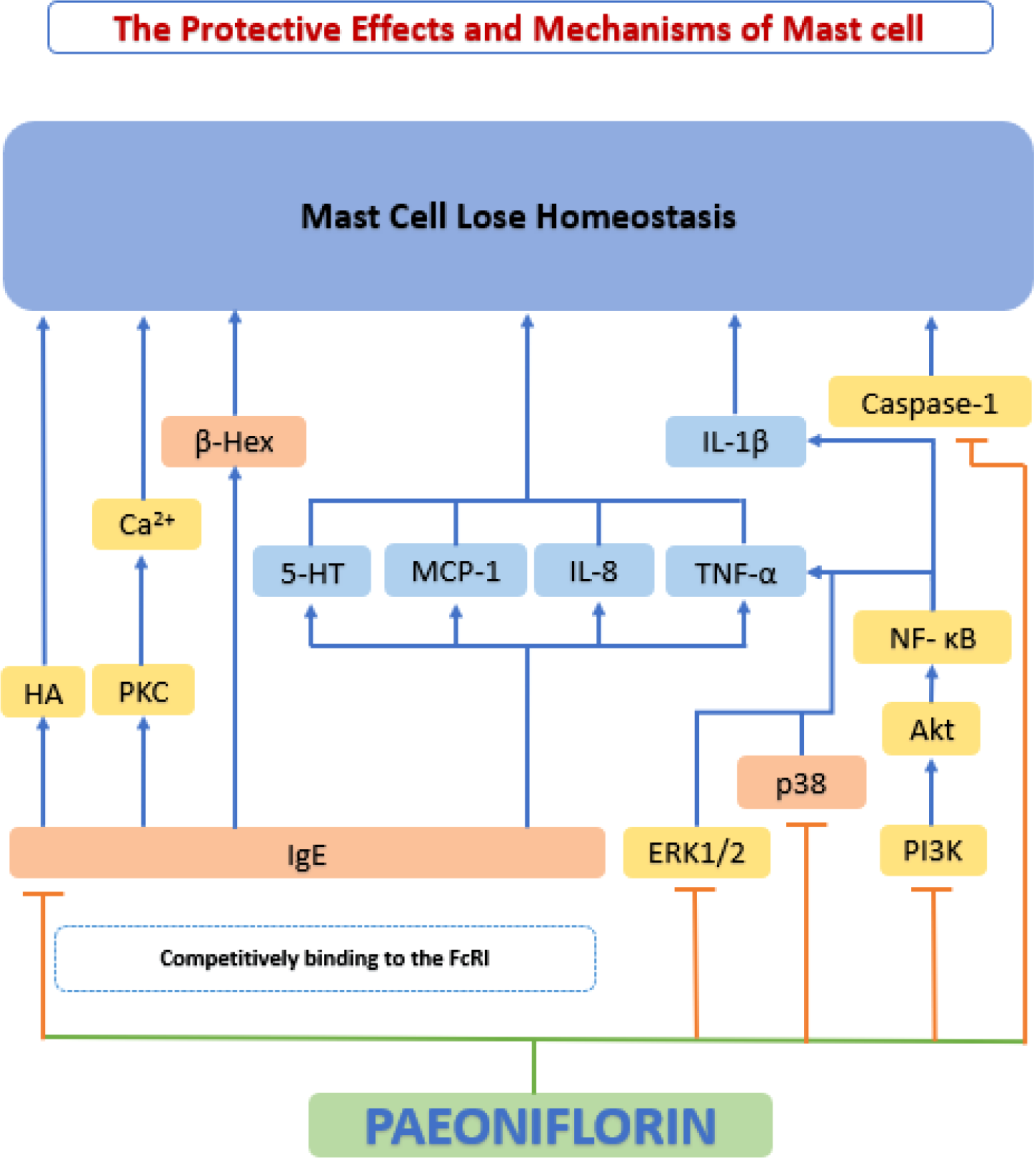
The protective effects and mechanisms of paeoniflorin on mast cell. The stabilizing effect of paeoniflorin on mast cells appears to be a promising new mechanism for the inhibition of atherosclerosis. 5-HT, 5-hydroxytryptamine; Akt, serine-threonine kinase; Ca2+, calcium2+; FcRI, high-affinity receptors for IgE; HA, histamine; IL-8, interleukin 8; IL-1β, interleukin 1β; MCP-1, monocyte chemoattractant protein-1; NF-κB, the nuclear factor-kB; PI3K, phosphatidylinositol-4,5-bisphosphate 3-kinase; PKC, protein kinase C.

Wang et al. (205) investigated phorbol 12-myristate 13-acetate plus calcium ionophore A23187 (PMACI)-induced HMC-1 cells and found that pre-processing with paeoniflorin significantly attenuated PMACI-induced TNF-α and IL-1β production and significantly inhibited PMACI-induced histamine (HA) release and caspase-1 activation in HMC-1 cells. Further studies showed that the mechanism of the above effects was that paeoniflorin inhibited the activation of NF-κB and MAPK signaling pathways. Zhao et al. (206) designed experiments following two stimulation pathways (IgE-dependent and IgE-independent stimulation) induced by RBL-2H3 cells and showed that paeoniflorin may inhibit the release of HA and β-hexosaminidase (β-Hex) by regulating intracellular Ca^2+^ concentration in mast cells, while paeoniflorin can regulate local inflammation through MAPK and PI3K/Akt pathways to regulate local inflammation. In addition, paeoniflorin can regulate mast cell proliferation and apoptosis by modulating NF-κB activity. Thus, the stabilizing effect of paeoniflorin on mast cells appears to be a promising new mechanism for the inhibition of atherosclerosis (**Figure 10**).

#### 4.6.2 Improvement of lipid metabolism

Impaired lipid metabolism plays a fundamental role in the pathogenesis of atherosclerosis (207). The liver working as the largest metabolic organ in the body took on the assignment in lipid metabolism by influencing lipid production, lipolysis, and serum lipoprotein uptake and secretion. Many clinical and experimental researches have shown that dysfunction of hepatic lipid metabolism is closely associated with the onset of atherosclerosis (208). Recent studies have shown that the Hydroxyproline-O-galactosyltransferase (GALT)2-angiogenin-like protein 3-lipoprotein lipase (GALT2-AngPTL3-LPL) pathway affects lipid metabolism from three aspects. Firstly, GALT2 directly regulates HDL metabolism of mammalian (209); secondly, GALT2 act as an inhibitor of ANGPTL3, which causes dyslipidemia (210); thirdly, LPL activity is controlled by ANGPTL3, and LPL is a key enzyme in lipid metabolism (211). Yang et al. (212) studied lipid metabolism in Wistar hyperlipidemic rats and showed that oral administration of paeoniflorin significantly reduced cholesterol, plasma triglyceride, and LDL levels and increased HDL levels. Xiao et al. (213) further explored the possible mechanisms using ApoE^-/-^ mice. Their study showed that paeoniflorin elevated plasma HDL-cholesterol levels and decreased plasma concentrations of LDL-cholesterol, total cholesterol, triglycerides, malondialdehyde, and 8-isoprostane by downregulating the expression of ANGPTL3 and upregulating the expression of GALT2 and LPL, alleviating dyslipidemia in mice. A study conducted by Li et al. (214) using SD rats also demonstrated that paeoniflorin reversed fructose-induced insulin resistance and hepatic steatosis, thereby inhibiting adipogenesis.

From the point of view of cholesterol metabolism, the level of cholesterol in circulating plasma is the result of the balance of various factors and its level depends mainly on intestinal absorption, hepatic synthesis and the degree of hepatic conversion to bile acids (215). In this process, 3-hydroxy-3-methylglutaryl-coenzyme A reductase (HMGCR) is one of the most key enzymes involved in cholesterol biosynthesis in living organisms (216). When cholesterol synthesis decreases, cells begin to deplete stored cholesterol and the number of low-density lipoprotein receptors (LDLR) increases, as LDLR plays a crucial role in cholesterol endocytosis, leading to a decrease in TC and LDL-C levels and affecting other lipid metabolic processes (217). Peroxisome proliferator-activated receptor (PPAR)-α plays a critical role in the pathogenesis of hepatic steatosis and enhances hepatic clearance of excess cholesterol (218). CYP7A1 is also closely associated with cholesterol metabolism, as it plays an instrumental role in bile acid metabolism (219). Hu et al. (220) found in the SD rat model of hyperlipidemia that oral administration of paeoniflorin significantly reduced serum levels of TC, LDLC, free cholesterol (FC), and cholesteryl ester (CE) in hyperlipidemic rats, while decreasing HMG-CoAR activity and upregulating the expression of LDLR, PPAR-α, and CYP7A1, effectively regulated hepatic cholesterol synthesis and metabolism. In addition, paeoniflorin also significantly increased hepatic SOD levels, decreased hepatic MDA levels, increased serum NO concentration and NOS activity, and protected liver from oxidative stress. PPARγ-LXRα-ABCA-1 is an important regulatory pathway for cholesterol export (221). PPARγ agonists induce ABCAl transcription (222). Oxidized cholesterol also activates LXR to induce ABCAl transcription; and LXRα transcription is also influenced by PPARγ (223). Zhang et al. (224) conducted experiment using C57BL/6J mice and found that paeoniflorin can lower cholesterol and improve lipid metabolism by inhibiting the PPARγ-LXRα-ABCA-1 pathway.

**Figure 11:**
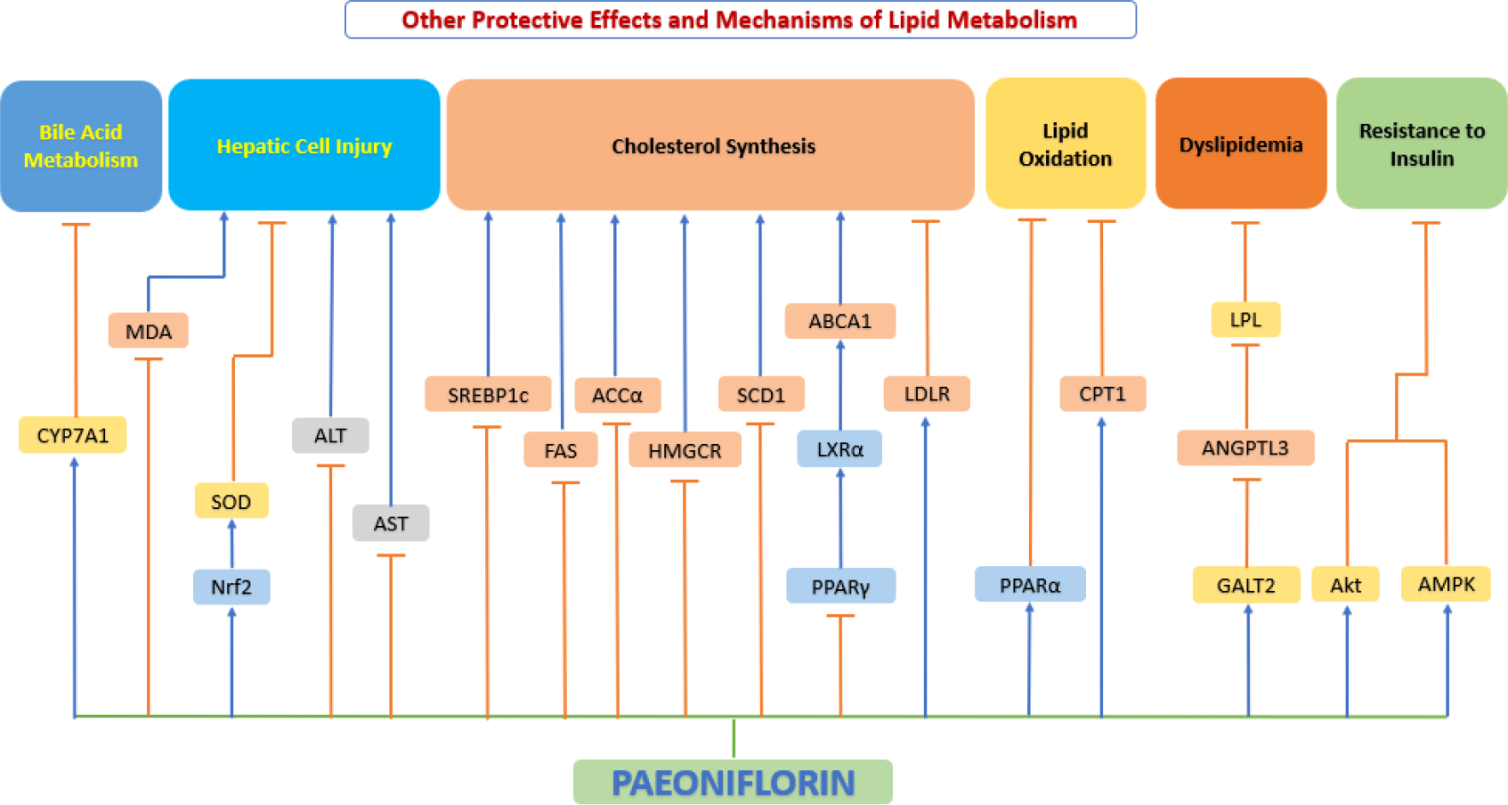
Other protective effects and mechanisms of paeoniflorin. Paeoniflorin can exert important anti-atherosclerotic effects by regulating lipid metabolism. ABCA1, ATP binding cassette transporter A1; ACCα, acetyl-CoA carboxylase-α; Akt, serine-threonine kinase; ALT, alanine aminotransferase; AMPK, AMP-activated protein kinase; ANGPTL3, Angiopoietin-like 3; AST, aspartate aminotransferase; CPT1, carnitine palmitoyltransferase 1; CYP7A1, cytochrome P450 7A1; FAS, fatty acid synthase; GALT2, Hydroxyproline-O-galactosyltransferase; HMGCR, anti-hydroxy-3-methylglutaryl-CoA reductase; LDLR, low-density lipoprotein receptor; LPL, lipoprotein lipase; LXRα, liver X receptor α; MDA, malondialdehyde; Nrf2, nuclear factor E2-related factor 2; PPAR−α, peroxisome proliferator−activated receptor α; PPAR−γ, peroxisome proliferator−activated receptor γ; SCD1, stearoyl-CoA desaturase 1; SOD, superoxide dismutase; SREBP1c, sterol regulatory element-binding protein 1c.

The above evidence suggests that paeoniflorin can significantly suppress serum levels of “Negative cholesterol” (such as LDL) and increase levels of “Positive cholesterol” (such as HDL), thereby regulating lipid metabolism and playing a critical role in inhibiting the development and progression of atherosclerosis (**Figure 11**).

## 5 Concluding remarks and future perspectives

Paeoniflorin has numerous protective effects in the development of atherosclerosis. The many protective effects can be attributed to the following mechanisms, such as anti-inflammatory/oxidant/recruitment/activation/proliferation/migration/apoptosis, adhesion regulation, homeostatic regulation, control of lipid metabolism, and regulation of vascular regeneration. All of these actions are based on different pathways and interact with each other.

### 5.1 Summary of the direct molecular targets of paeoniflorin

The molecular targets of paeoniflorin sorted out in this paper are very complicated, and the link between many various targets and signaling pathways (synergistic and antagonistic effects) cannot be clearly explained yet. For some cellular effects, current studies have only observed macroscopic results, e.g., cell migration, aggregation, foaming, etc., and the molecular mechanisms and gene targets behind them have not been studied in depth. Previous studies have found that some of the pharmacological effects of paeoniflorin are achieved possibly through: (1) regulation of the inflammatory response of immune cells through the balance of Th17 and Treg; (2) acts on HLA-DR and CD80 to regulate antigen presentation in the innate immunity function; (3) activating the LXR pathway, which regulates cholesterol metabolism and thus exerting anti-atherosclerotic effects; (4) regulates the inflammatory response of relevant cells and maintains cellular homeostasis through NF-kB, JNK, and ERK signaling pathways; (5) regulation of cell recruitment and adhesion by acting on ICAM-1 and VCAM-1 targets; (6) modulating the survival and fate of atherosclerosis-associated cells by acting on Bcl-2 and Bax targets; (7) regulation of relevant cellular phenotypic changes through STAT and TLR targets; (8) plays an important role against cellular oxidative stress through NO and SOD.

### 5.2 Drug ability and lead optimization of paeoniflorin

The wide variety of actions of paeoniflorin fully demonstrates that it can play an essential role in the prevention and treatment of atherosclerosis and has a large potential for clinical applications. Drug similarity is a very important aspect in translational medicine, and we examined the drug similarity of paeoniflorin through the SwissADME website (225) and Lipinski parameters showed good drug similarity. A bioavailability Score of 0.55 shows that paeoniflorin is highly worthy of further development as a new drug.

The oral bioavailability ensures the effective drug concentration achieved physiologically (226). As we presented in the previous pharmacokinetic content, the low permeability may be one of the direct factors for the relatively low bioavailability of paeoniflorin (21). Meanwhile, the lack of lipophilicity and the efflux effect (p-glycoprotein-mediated) may be another reason for the low permeability of paeoniflorin (18). The low bioavailability of paeoniflorin due to poor permeability, efflux of transporter proteins, and degradation by intestinal lumen hydrolysis greatly limit its clinical application, so pharmacologists have been working in various ways to optimize the drug.

One of the optimization paths is to improve the stability by structural modification of paeoniflorin. Paeoniflorin-6′-O-benzenesulfonate (also known as CP-25) is a new ester derivative of paeoniflorin developed by Wei et al. (227). It is mainly metabolized to paeoniflorin *in vivo* to exert pharmacological effects. Compared to paeoniflorin, the pharmacokinetics of CP-25 (in rats) reflects higher lipid solubility, resulting in superior absorption and distribution, while it has lower clearance and can stay in the body for longer periods of time, thus obtaining higher bioavailability (227). The development of paeoniflorin phospholipid complexes (228) and carrier micelle complexes (229) also greatly improved the lipophilicity and oral bioavailability of paeoniflorin. The pharmacological actions of paeoniflorin derivatives on atherosclerotic CVD remain to be investigated.

### 5.3 Animal experiments and clinical trials

Our review found that the protective effect of paeoniflorin against atherosclerosis has only been studied in rodents (mainly mice and rats). There are very few studies based on APOE^-/-^ and LDLR^-/-^ mice, that are currently the most mainstream animal models of atherosclerosis. However, there is a lack of validated studies in other large animal models, such as rabbit, quail, canines, porcine and monkeys.

However, until now, we still lack clinical data on paeoniflorin in CVD patients. Therefore, there is an urgent need for a multicenter, large-scale randomized design, placebo-controlled and blinded design clinical trial to evaluate the efficacy and safety of paeoniflorin based on a clinical perspective.

### 5.4 Potential effect of paeoniflorin on other risk factors associated with atherosclerosis

Two risk factors, hypertension and diabetes, have been relatively well studied. Relevant studies in epidemiology have shown that modifiable risk factors such as smoking, unhealthy diet, obesity and lack of physical activity, as well as underlying genetic factors, contribute to atherosclerosis (230). Numerous topical studies have further found that smoking induces oxidative stress, vascular inflammation, platelet clotting, and vascular dysfunction, which adversely affects the cardiovascular system (231). Dietary mediators and dietary habits in different regions also have an impact on atherosclerosis (232). Abnormal lipid metabolism, insulin resistance, inflammation, endothelial dysfunction, adipokine imbalance, and inflammatory vesicle activation, all of which are thought to underlie the relationship between unhealthy eating habits/obesity and atherosclerosis (233). Studies on sedentary lifestyle (physical inactivity) and cardiovascular events have shown that endothelial dysfunction due to reactive oxygen species may induce atherosclerosis (234). These risk factors are likely to be affected by paeoniflorin, further studies are warranted to elucidate whether paeoniflorin can prevent CVD by attenuating these risk factor associated vascular cell dysfunction.

In recent decades, the research of botanical drugs has made many remarkable achievements. The bioactive ingredients of some traditional herbal medicines have been developed into new drugs for clinical applications. Through literature review, we found that paeoniflorin can exert anti-atherosclerotic effects through multiple pathways and mechanisms, as well as possible synergistic therapeutic effects for some accompanying other chronic conditions (e.g., hypertension and diabetes), all of which highlight the advantages of paeoniflorin as a potential cardiovascular drug for further clinical development.

## Author contributions

Conceptualization: WY, SX, HY. Write: WY. Revise: WY, SX, II, XH, HY. All authors contributed to the article and approved the submitted version.

## References

[1] Garcia MC Rossen LM Bastian B Faul M Dowling NF Thomas CC. Potentially excess deaths from the five leading causes of death in metropolitan and nonmetropolitan counties - united states, 2010-2017. MMWR Surveill Summ (2019) 68(10):1–11. doi: 10.15585/mmwr.ss6810a1 31697657

[2] Sluimer JC Daemen MJ. Novel concepts in atherogenesis: angiogenesis and hypoxia in atherosclerosis. J Pathol (2009) 218(1):7–29. doi: 10.1002/path.2518 19309025

[3] Kim MJ Jung SK. Nutraceuticals for prevention of atherosclerosis: Targeting monocyte infiltration to the vascular endothelium. J Food Biochem (2020) 44(6):e13200. doi: 10.1111/jfbc.13200 32189369

[4] Tabas I García-Cardeña G Owens GK. Recent insights into the cellular biology of atherosclerosis. J Cell Biol (2015) 209(1):13–22. doi: 10.1083/jcb.201412052 25869663 PMC4395483

[5] Falk E. Pathogenesis of atherosclerosis. J Am Coll Cardiol (2006) 47(8 Suppl):C7–12. doi: 10.1016/j.jacc.2005.09.068 16631513

[6] Gisterå A Hansson GK. The immunology of atherosclerosis. Nat Rev Nephrol (2017) 13(6):368–80. doi: 10.1038/nrneph.2017.51 28392564

[7] Ferraro R Latina JM Alfaddagh A Michos ED Blaha MJ Jones SR. Evaluation and management of patients with stable angina: Beyond the ischemia paradigm: JACC state-of-the-Art review. J Am Coll Cardiol (2020) 76(19):2252–66. doi: 10.1016/j.jacc.2020.08.078 33153586

[8] Singh M Bedi US. Is atherosclerosis regression a realistic goal of statin therapy and what does that mean? Curr Atheroscler Rep (2013) 15(1):294. doi: 10.1007/s11883-012-0294-4 23250630

[9] Antons KA Williams CD Baker SK Phillips PS. Clinical perspectives of statin-induced rhabdomyolysis. Am J Med (2006) 119(5):400–9. doi: 10.1016/j.amjmed.2006.02.007 16651050

[10] Yu W Ilyas I Aktar N Xu S. A review on therapeutical potential of paeonol in atherosclerosis. Front Pharmacol (2022) 13:950337. doi: 10.3389/fphar.2022.950337 35991897 PMC9385965

[11] Jiao F Varghese K Wang S Liu Y Yu H Booz GW. Recent insights into the protective mechanisms of paeoniflorin in neurological, cardiovascular, and renal diseases. J Cardiovasc Pharmacol (2021) 77(6):728–34. doi: 10.1097/FJC.0000000000001021 PMC8169546 34001724

[12] Zhang L Wei W. Anti-inflammatory and immunoregulatory effects of paeoniflorin and total glucosides of paeony. Pharmacol Ther (2020) 207:107452. doi: 10.1016/j.pharmthera.2019.107452 31836457

[13] Tanaka T Kataoka M Tsuboi N Kouno I. New monoterpene glycoside esters and phenolic constituents of paeoniae radix, and increase of water solubility of proanthocyanidins in the presence of paeoniflorin. Chem Pharm Bull (Tokyo) (2000) 48(2):201–7. doi: 10.1248/cpb.48.201 10705504

[14] Li P Shen J Wang Z Liu S Liu Q Li Y. Genus paeonia: A comprehensive review on traditional uses, phytochemistry, pharmacological activities, clinical application, and toxicology. J Ethnopharmacol (2021) 269:113708. doi: 10.1016/j.jep.2020.113708 33346027

[15] Commission NP. Chinese Pharmacopoeia-baishao, in part I. Beijing: China Medical Science and Technology Press (2020) p. 108–9.

[16] Commission NP. Chinese Pharmacopoeia-chishao, in part I. Beijing: China Medical Science and Technology Press (2020) p. 165–6.

[17] Jiang H Li J Wang L Wang S Nie X Chen Y. Total glucosides of paeony: A review of its phytochemistry, role in autoimmune diseases, and mechanisms of action. J Ethnopharmacol (2020) 258:112913. doi: 10.1016/j.jep.2020.112913 32371143

[18] Zhou YX Gong XH Zhang H Peng C. A review on the pharmacokinetics of paeoniflorin and its anti-inflammatory and immunomodulatory effects. BioMed Pharmacother (2020) 130:110505. doi: 10.1016/j.biopha.2020.110505 32682112

[19] Ke ZC Cheng XL Lin CY Feng L Jia XB. [Characterization of oil/water partition coefficient of chishao terpene glucoside components based on contribution rate of representative components for myocardial ischemia]. Zhongguo Zhong Yao Za Zhi (2020) 45(16):3852–6. doi: 10.19540/j.cnki.cjcmm.20200622.302 32893580

[20] Hattori M Shu YZ Shimizu M Hayashi T Morita N Kobashi K. Metabolism of paeoniflorin and related compounds by human intestinal bacteria. Chem Pharm Bull (Tokyo) (1985) 33(9):3838–46. doi: 10.1248/cpb.33.3838 4092282

[21] Liu ZQ Jiang ZH Liu L Hu M. Mechanisms responsible for poor oral bioavailability of paeoniflorin: Role of intestinal disposition and interactions with sinomenine. Pharm Res (2006) 23(12):2768–80. doi: 10.1007/s11095-006-9100-8 17063398

[22] Chen Y Wang J Yuan L Zhou L Jia X Tan X. Interaction of the main components from the traditional Chinese drug pair chaihu-shaoyao based on rat intestinal absorption. Molecules (2011) 16(11):9600–10. doi: 10.3390/molecules16119600 PMC6264397 22095024

[23] Takeda S Isono T Wakui Y Matsuzaki Y Sasaki H Amagaya S. Absorption and excretion of paeoniflorin in rats. J Pharm Pharmacol (1995) 47(12a):1036–40. doi: 10.1111/j.2042-7158.1995.tb03293.x 8932691

[24] Jiang N Zheng B Feng Y Yin L Liu Y Cao L. A pharmacokinetics-pharmacodynamics study of single-dose total glucosides of paeony capsule on reducing serum total bile acid in hepatic injury rats. Pharm Biol (2021) 59(1):769–77. doi: 10.1080/13880209.2021.1937232 PMC8218697 34152236

[25] Hu PY Liu D Zheng Q Wu Q Tang Y Yang M. Elucidation of transport mechanism of paeoniflorin and the influence of ligustilide, senkyunolide I and senkyunolide a on paeoniflorin transport through mdck-Mdr1 cells as blood-brain barrier in vitro model. Molecules (2016) 21(3):300. doi: 10.3390/molecules21030300 26950101 PMC6273373

[26] Liu ZQ Zhou H Liu L Jiang ZH Wong YF Xie Y. Influence of co-administrated sinomenine on pharmacokinetic fate of paeoniflorin in unrestrained conscious rats. J Ethnopharmacol (2005) 99(1):61–7. doi: 10.1016/j.jep.2005.01.052 15848021

[27] Li X Shi F Zhang R Sun C Gong C Jian L. Pharmacokinetics, safety, and tolerability of amygdalin and paeoniflorin after single and multiple intravenous infusions of huoxue-tongluo lyophilized powder for injection in healthy Chinese volunteers. Clin Ther (2016) 38(2):327–37. doi: 10.1016/j.clinthera.2015.12.005 26749220

[28] Ke ZC Yang N Hou XF Wang AD Feng L Jia XB. [Metabolism of paeoniflorin by rat intestinal flora in vitro]. Zhongguo Zhong Yao Za Zhi (2016) 41(20):3839–45. doi: 10.4268/cjcmm20162021 28929664

[29] Zhu L Sun S Hu Y Liu Y. Metabolic study of paeoniflorin and total paeony glucosides from paeoniae radix rubra in rats by high-performance liquid chromatography coupled with sequential mass spectrometry. BioMed Chromatogr (2018) 32(4). doi: 10.1002/bmc.4141 29148594

[30] Chobanian AV. Single risk factor intervention may be inadequate to inhibit atherosclerosis progression when hypertension and hypercholesterolemia coexist. Hypertension (1991) 18(2):130–1. doi: 10.1161/01.hyp.18.2.130 1885221

[31] Michel JB Martin-Ventura JL. Red blood cells and hemoglobin in human atherosclerosis and related arterial diseases. Int J Mol Sci (2020) 21(18). doi: 10.3390/ijms21186756 PMC7554753 32942605

[32] Sugiyama D Okamura T Watanabe M Higashiyama A Okuda N Nakamura Y. Risk of hypercholesterolemia for cardiovascular disease and the population attributable fraction in a 24-year Japanese cohort study. J Atheroscler Thromb (2015) 22(1):95–107. doi: 10.5551/jat.25908 25185893

[33] Davis NE. Atherosclerosis–an inflammatory process. J Insur Med (2005) 37(1):72–5. Available at: https://aaimedicine.org/journal-of-insurance-medicine/jim/2005/037-01-0072.pdf 15895704

[34] Li H Jiao Y Xie M. Paeoniflorin ameliorates atherosclerosis by suppressing TLR4-mediated NF-kappaB activation. Inflammation (2017) 40(6):2042–51. doi: 10.1007/s10753-017-0644-z 28791506

[35] Wu DF Jin SR Liu J Wu JH Xiao YH Hu S. Primary study on effects of paeoniflorin against experimental atherosclerosis in ApoE-/- mice. Chin Hosp Pharm J (2015) 35:385–8. doi: 10.13286/j.cnki.chinhosppharmacyj.2015.05.04

[36] Xu WP Wang YY Meng XW Wang Y. Effect of paeoniflorin on blood lipids and atherosclerotic plaque in mice with apolipoprotein e deficient. J Tianjin Univ Chin Med (2014) 33:210–2. doi: 10.11656/j.issn.1673-9043.2014.03.06

[37] Hurtubise J McLellan K Durr K Onasanya O Nwabuko D Ndisang JF. The different facets of dyslipidemia and hypertension in atherosclerosis. Curr Atheroscler Rep (2016) 18(12):82. doi: 10.1007/s11883-016-0632-z 27822682

[38] Wu J Zhang D Hu L Zheng X Chen C. Paeoniflorin alleviates NG-nitro-L-arginine methyl ester (L-NAME)-induced gestational hypertension and upregulates silent information regulator 2 related enzyme 1 (SIRT1) to reduce H2O2-induced endothelial cell damage. Bioengineered (2022) 13(2):2248–58. doi: 10.1080/21655979.2021.2024325 PMC8973614 35030965

[39] Poznyak A Grechko AV Poggio P Myasoedova VA Alfieri V Orekhov AN. The diabetes mellitus-atherosclerosis connection: The role of lipid and glucose metabolism and chronic inflammation. Int J Mol Sci (2020) 21(5). doi: 10.3390/ijms21051835 PMC7084712 32155866

[40] Jung HS. Clinical implications of glucose variability: Chronic complications of diabetes. Endocrinol Metab (Seoul) (2015) 30(2):167–74. doi: 10.3803/EnM.2015.30.2.167 PMC4508260 26194076

[41] Wang J Yin H Huang Y Guo C Xia C Liu Q. Panax quinquefolius saponin of stem and leaf attenuates intermittent high glucose-induced oxidative stress injury in cultured human umbilical vein endothelial cells via PI3K/Akt/GSK-3 β pathway. Evid Based Complement Alternat Med 2013 (2013) p:196283. doi: 10.1155/2013/196283 PMC3728514 23956765

[42] Risso A Mercuri F Quagliaro L Damante G Ceriello A. Intermittent high glucose enhances apoptosis in human umbilical vein endothelial cells in culture. Am J Physiol Endocrinol Metab (2001) 281(5):E924–30. doi: 10.1152/ajpendo.2001.281.5.E924 11595647

[43] Liu TS Pei YH Peng YP Chen J Jiang SS Gong JB. Oscillating high glucose enhances oxidative stress and apoptosis in human coronary artery endothelial cells. J Endocrinol Invest (2014) 37(7):645–51. doi: 10.1007/s40618-014-0086-5 24859911

[44] Liu T Gong J Chen Y Jiang S. Periodic vs constant high glucose in inducing pro-inflammatory cytokine expression in human coronary artery endothelial cells. Inflammation Res (2013) 62(7):697–701. doi: 10.1007/s00011-013-0623-2 23625043

[45] Wang JS Huang Y Zhang S Yin HJ Zhang L Zhang YH. A protective role of paeoniflorin in fluctuant hyperglycemia-induced vascular endothelial injuries through antioxidative and anti-inflammatory effects and reduction of PKCbeta1. Oxid Med Cell Longev (2019) 2019:5647219. doi: 10.1155/2019/5647219 31093316 PMC6481012

[46] Weber C Noels H. Atherosclerosis: current pathogenesis and therapeutic options. Nat Med (2011) 17(11):1410–22. doi: 10.1038/nm.2538 22064431

[47] Xu S. Endothelial dysfunction in atherosclerotic cardiovascular diseases and beyond: From mechanism to pharmacotherapies. Pharmacol Rev (2021) 73(3):924–67. doi: 10.1124/pharmrev.120.000096 34088867

[48] Gimbrone MA Jr. García-Cardeña G. Endothelial cell dysfunction and the pathobiology of atherosclerosis. Circ Res (2016) 118(4):620–36. doi: 10.1161/CIRCRESAHA.115.306301 PMC4762052 26892962

[49] Habas K Shang L. Alterations in intercellular adhesion molecule 1 (ICAM-1) and vascular cell adhesion molecule 1 (VCAM-1) in human endothelial cells. Tissue Cell (2018) 54:139–43. doi: 10.1016/j.tice.2018.09.002 30309503

[50] Mehta D Malik AB. Signaling mechanisms regulating endothelial permeability. Physiol Rev (2006) 86(1):279–367. doi: 10.1152/physrev.00012.2005 16371600

[51] Lai CH Kuo KH Leo JM. Critical role of actin in modulating BBB permeability. Brain Res Brain Res Rev (2005) 50(1):7–13. doi: 10.1016/j.brainresrev.2005.03.007 16291072

[52] Blaine J Dylewski J. Regulation of the actin cytoskeleton in podocytes. Cells (2020) 9(7). doi: 10.3390/cells9071700 PMC7408282 32708597

[53] Ghosh M Song X Mouneimne G Sidani M Lawrence DS Condeelis JS. Cofilin promotes actin polymerization and defines the direction of cell motility. Science (2004) 304(5671):743–6. doi: 10.1126/science.1094561 15118165

[54] Xu H Song J Gao X Xu Z Xu X Xia Y. Paeoniflorin attenuates lipopolysaccharide-induced permeability of endothelial cells: involvements of f-actin expression and phosphorylations of PI3K/Akt and PKC. Inflammation (2013) 36(1):216–25. doi: 10.1007/s10753-012-9537-3 23053726

[55] Zhang Q Liu J Duan H Li R Peng W Wu C. Activation of Nrf2/HO-1 signaling: An important molecular mechanism of herbal medicine in the treatment of atherosclerosis via the protection of vascular endothelial cells from oxidative stress. J Adv Res (2021) 34:43–63. doi: 10.1016/j.jare.2021.06.023 35024180 PMC8655139

[56] Li Q Youn JY Cai H. Mechanisms and consequences of endothelial nitric oxide synthase dysfunction in hypertension. J Hypertens (2015) 33(6):1128–36. doi: 10.1097/HJH.0000000000000587 PMC4816601 25882860

[57] Fu BD Liu YE Zhang XX Zhou YW. Effects of paeoniflorin on the production of NO, eNOS and CAMs in cultured human umbilical vein endothelial cells under hypoxia. Chin J Pharm Anal (2007) 27:555–7. doi: 10.16155/j.0254-1793.2007.04.011

[58] Cini M Fariello RG Bianchetti A Moretti A. Studies on lipid peroxidation in the rat brain. Neurochem Res (1994) 19(3):283–8. doi: 10.1007/BF00971576 8177367

[59] Ryter SW Kim HP Hoetzel A Park JW Nakahira K Wang X. Mechanisms of cell death in oxidative stress. Antioxid Redox Signal (2007) 9(1):49–89. doi: 10.1089/ars.2007.9.49 17115887

[60] Yu J Zhu X Qi X Che J Cao B. Paeoniflorin protects human EA.hy926 endothelial cells against gamma-radiation induced oxidative injury by activating the NF-E2-related factor 2/heme oxygenase-1 pathway. Toxicol Lett (2013) 218(3):224–34. doi: 10.1016/j.toxlet.2013.01.028 23403272

[61] Brookes PS Yoon Y Robotham JL Anders MW Sheu SS. Calcium, ATP, and ROS: a mitochondrial love-hate triangle. Am J Physiol Cell Physiol (2004) 287(4):C817–33. doi: 10.1152/ajpcell.00139.2004 15355853

[62] Peng W Cai G Xia Y Chen J Wu P Wang Z. Mitochondrial dysfunction in atherosclerosis. DNA Cell Biol (2019) 38(7):597–606. doi: 10.1089/dna.2018.4552 31095428

[63] Song S Xiao X Guo D Mo L Bu C Ye W. Protective effects of paeoniflorin against AOPP-induced oxidative injury in HUVECs by blocking the ROS-HIF-1alpha/VEGF pathway. Phytomedicine (2017) 34:115–26. doi: 10.1016/j.phymed.2017.08.010 28899493

[64] Jiang J Dong C Zhai L Lou J Jin J Cheng S. Paeoniflorin suppresses TBHP-induced oxidative stress and apoptosis in human umbilical vein endothelial cells via the Nrf2/HO-1 signaling pathway and improves skin flap survival. Front Pharmacol (2021) 12:735530. doi: 10.3389/fphar.2021.735530 34803685 PMC8600365

[65] Erpapazoglou Z Mouton-Liger F Corti O. From dysfunctional endoplasmic reticulum-mitochondria coupling to neurodegeneration. Neurochem Int (2017) 109:171–83. doi: 10.1016/j.neuint.2017.03.021 28389271

[66] Giorgio V Soriano ME Basso E Bisetto E Lippe G Forte MA. Cyclophilin d in mitochondrial pathophysiology. Biochim Biophys Acta (2010) 1797(6-7):1113–8. doi: 10.1016/j.bbabio.2009.12.006 PMC2888675 20026006

[67] Walter P Ron D. The unfolded protein response: from stress pathway to homeostatic regulation. Science (2011) 334(6059):1081–6. doi: 10.1126/science.1209038 22116877

[68] Harding HP Zhang Y Bertolotti A Zeng H Ron D. Perk is essential for translational regulation and cell survival during the unfolded protein response. Mol Cell (2000) 5(5):897–904. doi: 10.1016/S1097-2765(00)80330-5 10882126

[69] Ou T-T Chuang C-M Leung Y-M Lee IT Wu C-H. Paeoniflorin attenuates oxidative stress injury and improves mitochondrial membrane potential in human EA.hy926 endothelial cell through p-eIF2α and CHOP signaling. J Funct Foods (2021) 86. doi: 10.1016/j.jff.2021.104676

[70] Deng M Tang Y Li W Wang X Zhang R Zhang X. The endotoxin delivery protein HMGB1 mediates caspase-11-Dependent lethality in sepsis. Immunity (2018) 49(4):740–753.e7. doi: 10.1016/j.immuni.2018.08.016 30314759 PMC6300139

[71] Davison G Gleeson M Phillips S. Antioxidant supplementation and immunoendocrine responses to prolonged exercise. Med Sci Sports Exerc (2007) 39(4):645–52. doi: 10.1249/mss.0b013e318031303d 17414802

[72] Soysal P Arik F Smith L Jackson SE Isik AT. Inflammation, frailty and cardiovascular disease. Adv Exp Med Biol 2020 (1216) p:55–64. doi: 10.1007/978-3-030-33330-0_7 31894547

[73] Groh L Keating ST Joosten LAB Netea MG Riksen NP. Monocyte and macrophage immunometabolism in atherosclerosis. Semin Immunopathol (2018) 40(2):203–14. doi: 10.1007/s00281-017-0656-7 PMC5809534 28971272

[74] Bi Y Han X Lai Y Fu Y Li K Zhang W. Systems pharmacological study based on UHPLC-Q-Orbitrap-HRMS, network pharmacology and experimental validation to explore the potential mechanisms of danggui-Shaoyao-San against atherosclerosis. J Ethnopharmacol (2021) 278:114278. doi: 10.1016/j.jep.2021.114278 34087397

[75] Iwakoshi NN Lee AH Vallabhajosyula P Otipoby KL Rajewsky K Glimcher LH. Plasma cell differentiation and the unfolded protein response intersect at the transcription factor XBP-1. Nat Immunol (2003) 4(4):321–9. doi: 10.1038/ni907 12612580

[76] Schmitz G Ruebsaamen K. Metabolism and atherogenic disease association of lysophosphatidylcholine. Atherosclerosis (2010) 208(1):10–8. doi: 10.1016/j.atherosclerosis.2009.05.029 19570538

[77] Lee W Kim TH Ku SK Min KJ Lee HS Kwon TK. Barrier protective effects of withaferin a in HMGB1-induced inflammatory responses in both cellular and animal models. Toxicol Appl Pharmacol (2012) 262(1):91–8. doi: 10.1016/j.taap.2012.04.025 22561332

[78] Luan ZG Zhang H Yang PT Ma XC Zhang C Guo RX. HMGB1 activates nuclear factor-κB signaling by RAGE and increases the production of TNF-α in human umbilical vein endothelial cells. Immunobiology (2010) 215(12):956–62. doi: 10.1016/j.imbio.2009.11.001 20163887

[79] Li JZ Wu JH Yu SY Shao QR Dong XM. Inhibitory effects of paeoniflorin on lysophosphatidylcholine-induced inflammatory factor production in human umbilical vein endothelial cells. Int J Mol Med (2013) 31(2):493–7. doi: 10.3892/ijmm.2012.1211 23241947

[80] Wang Y Huang G Vogel P Neale G Reizis B Chi H. Transforming growth factor beta-activated kinase 1 (TAK1)-dependent checkpoint in the survival of dendritic cells promotes immune homeostasis and function. Proc Natl Acad Sci U.S.A. (2012) 109(6):E343–52. doi: 10.1073/pnas.1115635109 PMC3277515 22308391

[81] Yu M Wu X Wang J He M Han H Hu S. Paeoniflorin attenuates monocrotaline-induced pulmonary arterial hypertension in rats by suppressing TAK1-MAPK/NF-kappaB pathways. Int J Med Sci (2022) 19(4):681–94. doi: 10.7150/ijms.69289 PMC9108400 35582418

[82] Hope SA Meredith IT. Cellular adhesion molecules and cardiovascular disease. part i. their expression and role in atherogenesis. Intern Med J (2003) 33(8):380–6. doi: 10.1046/j.1444-0903.2003.00378.x 12895171

[83] Adrielle Lima Vieira R Nascimento de Freitas R Volp AC. Adhesion molecules and chemokines; relation to anthropometric, body composition, biochemical and dietary variables. Nutr Hosp (2014) 30(2):223–36. doi: 10.3305/nh.2014.30.2.7416 25208773

[84] Choi EY Chavakis E Czabanka MA Langer HF Fraemohs L Economopoulou M. Del-1, an endogenous leukocyte-endothelial adhesion inhibitor, limits inflammatory cell recruitment. Science (2008) 322(5904):1101–4. doi: 10.1126/science.1165218 PMC2753175 19008446

[85] Sumagin R Sarelius IH. A role for ICAM-1 in maintenance of leukocyte-endothelial cell rolling interactions in inflamed arterioles. Am J Physiol Heart Circ Physiol (2007) 293(5):H2786–98. doi: 10.1152/ajpheart.00720.2007 17704289

[86] Cybulsky MI Gimbrone MA Jr. Endothelial expression of a mononuclear leukocyte adhesion molecule during atherogenesis. Science (1991) 251(4995):788–91. doi: 10.1126/science.1990440 1990440

[87] Antoine M Tag CG Gressner AM Hellerbrand C Kiefer P. Expression of e-selectin ligand-1 (CFR/ESL-1) on hepatic stellate cells: implications for leukocyte extravasation and liver metastasis. Oncol Rep (2009) 21(2):357–62. doi: 10.3892/or_00000230 19148508

[88] Lawson C Wolf S. ICAM-1 signaling in endothelial cells. Pharmacol Rep (2009) 61(1):22–32. doi: 10.1016/S1734-1140(09)70004-0 19307690

[89] Li W Ishihara K Yokota T Nakagawa T Koyama N Jin J. Reduced alpha4beta1 integrin/VCAM-1 interactions lead to impaired pre-b cell repopulation in alpha 1,6-fucosyltransferase deficient mice. Glycobiology (2008) 18(1):114–24. doi: 10.1093/glycob/cwm107 17913729

[90] Hughes CE Nibbs RJB. A guide to chemokines and their receptors. FEBS J (2018) 285(16):2944–71. doi: 10.1111/febs.14466 PMC6120486 29637711

[91] Deshmane SL Kremlev S Amini S Sawaya BE. Monocyte chemoattractant protein-1 (MCP-1): an overview. J Interferon Cytokine Res (2009) 29(6):313–26. doi: 10.1089/jir.2008.0027 PMC2755091 19441883

[92] Wang J Zhou JY Wu GS. ERK-dependent MKP-1-mediated cisplatin resistance in human ovarian cancer cells. Cancer Res (2007) 67(24):11933–41. doi: 10.1158/0008-5472.CAN-07-5185 18089824

[93] Huber J Fürnkranz A Bochkov VN Patricia MK Lee H Hedrick CC. Specific monocyte adhesion to endothelial cells induced by oxidized phospholipids involves activation of cPLA2 and lipoxygenase. J Lipid Res (2006) 47(5):1054–62. doi: 10.1194/jlr.M500555-JLR200 16461778

[94] Covert MW Leung TH Gaston JE Baltimore D. Achieving stability of lipopolysaccharide-induced NF-kappaB activation. Science (2005) 309(5742):1854–7. doi: 10.1126/science.1112304 16166516

[95] Collins T Read MA Neish AS Whitley MZ Thanos D Maniatis T. Transcriptional regulation of endothelial cell adhesion molecules: NF-kappa b and cytokine-inducible enhancers. FASEB J (1995) 9(10):899–909. doi: 10.1096/fasebj.9.10.7542214 7542214

[96] Chen T Guo ZP Wang L Qin S Cao N Li MM. Paeoniflorin suppresses vascular damage and the expression of e-selectin and ICAM-1 in a mouse model of cutaneous arthus reaction. Exp Dermatol (2013) 22(7):453–7. doi: 10.1111/exd.12174 23800055

[97] Jin L Zhang LM Xie KQ Ye Y Feng L. Paeoniflorin suppresses the expression of intercellular adhesion molecule-1 (ICAM-1) in endotoxin-treated human monocytic cells. Br J Pharmacol (2011) 164(2b):694–703. doi: 10.1111/j.1476-5381.2011.01464.x 21542832 PMC3188904

[98] Wang Y Che J Zhao H Tang J Shi G. Paeoniflorin attenuates oxidized low-density lipoprotein-induced apoptosis and adhesion molecule expression by autophagy enhancement in human umbilical vein endothelial cells. J Cell Biochem (2019) 120(6):9291–9. doi: 10.1002/jcb.28204 30548681

[99] Xu H Gao X Song J Wang F Xu Z Lu D. Peoniflorin prevents the adhesion between inflammatory endothelial cells and leukocytes through inhibiting the activation of MAPKs and NF-κB. Drug Dev Res (2010) 71(5):275–84. doi: 10.1002/ddr.20372

[100] Klionsky DJ Codogno P. The mechanism and physiological function of macroautophagy. J Innate Immun (2013) 5(5):427–33. doi: 10.1159/000351979 PMC6741458 23774579

[101] Gatica D Chiong M Lavandero S Klionsky DJ. Molecular mechanisms of autophagy in the cardiovascular system. Circ Res (2015) 116(3):456–67. doi: 10.1161/CIRCRESAHA.114.303788 PMC4313620 25634969

[102] Hu P Lai D Lu P Gao J He H. ERK and akt signaling pathways are involved in advanced glycation end product-induced autophagy in rat vascular smooth muscle cells. Int J Mol Med (2012) 29(4):613–8. doi: 10.3892/ijmm.2012.891 PMC3573741 22293957

[103] Mizushima N Yoshimori T. How to interpret LC3 immunoblotting. Autophagy (2007) 3(6):542–5. doi: 10.4161/auto.4600 17611390

[104] He C Klionsky DJ. Regulation mechanisms and signaling pathways of autophagy. Annu Rev Genet (2009) 43:67–93. doi: 10.1146/annurev-genet-102808-114910 19653858 PMC2831538

[105] Goldblatt ZE Cirka HA Billiar KL. Mechanical regulation of apoptosis in the cardiovascular system. Ann BioMed Eng (2021) 49(1):75–97. doi: 10.1007/s10439-020-02659-x 33169343 PMC7775273

[106] Zhang T Tian F Wang J Jing J Zhou SS Chen YD. Atherosclerosis-associated endothelial cell apoptosis by MiR-429-Mediated down regulation of bcl-2. Cell Physiol Biochem (2015) 37(4):1421–30. doi: 10.1159/000438511 26489013

[107] Chen F Eriksson P Kimura T Herzfeld I Valen G. Apoptosis and angiogenesis are induced in the unstable coronary atherosclerotic plaque. Coron Artery Dis (2005) 16(3):191–7. doi: 10.1097/00019501-200505000-00009 15818089

[108] Yue J López JM. Understanding MAPK signaling pathways in apoptosis. Int J Mol Sci (2020) 21(7). doi: 10.3390/ijms21072346 PMC7177758 32231094

[109] Ji Q Yang L Zhou J Lin R Zhang J Lin Q. Protective effects of paeoniflorin against cobalt chloride-induced apoptosis of endothelial cells via HIF-1alpha pathway. Toxicol In Vitro (2012) 26(3):455–61. doi: 10.1016/j.tiv.2012.01.016 22269387

[110] Greijer AE van der Wall E. The role of hypoxia inducible factor 1 (HIF-1) in hypoxia induced apoptosis. J Clin Pathol (2004) 57(10):1009–14. doi: 10.1136/jcp.2003.015032 PMC1770458 15452150

[111] Yu W Fu YC Chen CJ Wang X Wang W. SIRT1: a novel target to prevent atherosclerosis. J Cell Biochem (2009) 108(1):10–3. doi: 10.1002/jcb.22240 19562740

[112] Filomeni G De Zio D Cecconi F. Oxidative stress and autophagy: the clash between damage and metabolic needs. Cell Death Differ (2015) 22(3):377–88. doi: 10.1038/cdd.2014.150 PMC4326572 25257172

[113] Kang R Tang D Lotze MT Zeh HJ 3rd. RAGE regulates autophagy and apoptosis following oxidative injury. Autophagy (2011) 7(4):442–4. doi: 10.4161/auto.7.4.14681 21317562

[114] Chen Y Du X Zhou Y Zhang Y Yang Y Liu Z. Paeoniflorin protects HUVECs from AGE-BSA-induced injury via an autophagic pathway by acting on the RAGE. Int J Clin Exp Pathol (2015) 8(1):53–62. Available at: https://www.ncbi.nlm.nih.gov/pmc/articles/PMC4348843/pdf/ijcep0008-0053.pdf 25755692 PMC4348843

[115] Lin DT Lechleiter JD. Mitochondrial targeted cyclophilin d protects cells from cell death by peptidyl prolyl isomerization. J Biol Chem (2002) 277(34):31134–41. doi: 10.1074/jbc.M112035200 12077116

[116] Suzuki T Carrier EJ Talati MH Rathinasabapathy A Chen X Nishimura R. Isolation and characterization of endothelial-to-mesenchymal transition cells in pulmonary arterial hypertension. Am J Physiol Lung Cell Mol Physiol (2018) 314(1):L118–l126. doi: 10.1152/ajplung.00296.2017 28935639 PMC5866427

[117] Medici D. Endothelial-mesenchymal transition in regenerative medicine. Stem Cells Int 2016 (2016) p:6962801. doi: 10.1155/2016/6962801 PMC4838799 27143978

[118] Liu HT Zhou ZX Ren Z Yang S Liu LS Wang Z. EndMT: Potential target of H(2)S against atherosclerosis. Curr Med Chem (2021) 28(18):3666–80. doi: 10.2174/0929867327999201116194634 33200693

[119] Lacolley P Regnault V Segers P Laurent S. Vascular smooth muscle cells and arterial stiffening: Relevance in development, aging, and disease. Physiol Rev (2017) 97(4):1555–617. doi: 10.1152/physrev.00003.2017 28954852

[120] Qin C Liu Z. In atherogenesis, the apoptosis of endothelial cell itself could directly induce over-proliferation of smooth muscle cells. Med Hypotheses (2007) 68(2):275–7. doi: 10.1016/j.mehy.2006.07.037 17011140

[121] Labrecque L Lamy S Chapus A Mihoubi S Durocher Y Cass B. Combined inhibition of PDGF and VEGF receptors by ellagic acid, a dietary-derived phenolic compound. Carcinogenesis (2005) 26(4):821–6. doi: 10.1093/carcin/bgi024 15661805

[122] Chang Y Uen YH Chen CC Lin SC Tseng SY Wang YH. Platonin inhibited PDGF-BB-induced proliferation of rat vascular smooth muscle cells via JNK1/2-dependent signaling. Acta Pharmacol Sin (2011) 32(11):1337–44. doi: 10.1038/aps.2011.105 PMC4002726 21892199

[123] Lee CK Lee HM Kim HJ Park HJ Won KJ Roh HY. Syk contributes to PDGF-BB-mediated migration of rat aortic smooth muscle cells via MAPK pathways. Cardiovasc Res (2007) 74(1):159–68. doi: 10.1016/j.cardiores.2007.01.012 17303097

[124] Guo Y Zhao Y Li L Wei X Gao P Zhou Y. Concentrationdependent effects of paeoniflorin on proliferation and apoptosis of vascular smooth muscle cells. Mol Med Rep (2017) 16(6):9567–72. doi: 10.3892/mmr.2017.7776 29039520

[125] Li W Zhi W Liu F Zhao J Yao Q Niu X. Paeoniflorin inhibits VSMCs proliferation and migration by arresting cell cycle and activating HO-1 through MAPKs and NF-kappaB pathway. Int Immunopharmacol (2018) 54:103–11. doi: 10.1016/j.intimp.2017.10.017 29121532

[126] Chen JJ Zhang J Cai Y Zhou YB Wen GB Tang CS. C-type natriuretic peptide inhibiting vascular calcification might involve decreasing bone morphogenic protein 2 and osteopontin levels. Mol Cell Biochem (2014) 392(1-2):65–76. doi: 10.1007/s11010-014-2019-1 24710639

[127] Ortmann J Veit M Zingg S Santo Di S Traupe T Yang Z. Estrogen receptor-α but not -β or GPER inhibits high glucose-induced human VSMC proliferation: potential role of ROS and ERK. J Clin Endocrinol Metab (2011) 96(1):220–8. doi: 10.1210/jc.2010-0943 PMC3038487 20962025

[128] Collis MG Hourani SM. Adenosine receptor subtypes. Trends Pharmacol Sci (1993) 14(10):360–6. doi: 10.1016/0165-6147(93)90094-Z 8296392

[129] Fredholm BB Irenius E Kull B Schulte G. Comparison of the potency of adenosine as an agonist at human adenosine receptors expressed in Chinese hamster ovary cells. Biochem Pharmacol (2001) 61(4):443–8. doi: 10.1016/S0006-2952(00)00570-0 11226378

[130] Feoktistov I Ryzhov S Zhong H Goldstein AE Matafonov A Zeng D. Hypoxia modulates adenosine receptors in human endothelial and smooth muscle cells toward an A2B angiogenic phenotype. Hypertension (2004) 44(5):649–54. doi: 10.1161/01.HYP.0000144800.21037.a5 15452028

[131] Dubey RK Gillespie DG Mi Z Jackson EK. Adenosine inhibits growth of human aortic smooth muscle cells via A2B receptors. Hypertension (1998) 31(1 Pt 2):516–21. doi: 10.1161/01.HYP.31.1.516 9453355

[132] Qian G Cao J Chen C Wang L Huang X Ding C. Paeoniflorin inhibits pulmonary artery smooth muscle cells proliferation via upregulating A2B adenosine receptor in rat. PloS One (2013) 8(7):e69141. doi: 10.1371/journal.pone.0069141 23935939 PMC3728310

[133] Fan X Wu J Yang H Yan L Wang S. Paeoniflorin blocks the proliferation of vascular smooth muscle cells induced by plateletderived growth factorBB through ROS mediated ERK1/2 and p38 signaling pathways. Mol Med Rep (2018) 17(1):1676–82. doi: 10.3892/mmr.2017.8093 PMC5780110 29257209

[134] Grootaert MOJ Bennett MR. Vascular smooth muscle cells in atherosclerosis: time for a re-assessment. Cardiovasc Res (2021) 117(11):2326–39. doi: 10.1093/cvr/cvab046 PMC8479803 33576407

[135] Bennett MR Sinha S Owens GK. Vascular smooth muscle cells in atherosclerosis. Circ Res (2016) 118(4):692–702. doi: 10.1161/CIRCRESAHA.115.306361 26892967 PMC4762053

[136] Pedersen BK. Anti-inflammatory effects of exercise: role in diabetes and cardiovascular disease. Eur J Clin Invest (2017) 47(8):600–11. doi: 10.1111/eci.12781 28722106

[137] Wu H Zhao G Jiang K Chen X Zhu Z Qiu C. Plantamajoside ameliorates lipopolysaccharide-induced acute lung injury via suppressing NF-κB and MAPK activation. Int Immunopharmacol (2016) 35:315–22. doi: 10.1016/j.intimp.2016.04.013 27089391

[138] Grootaert MOJ Moulis M Roth L Martinet W Vindis C Bennett MR. Vascular smooth muscle cell death, autophagy and senescence in atherosclerosis. Cardiovasc Res (2018) 114(4):622–34. doi: 10.1093/cvr/cvy007 29360955

[139] Dong Y Chen H Gao J Liu Y Li J Wang J. Molecular machinery and interplay of apoptosis and autophagy in coronary heart disease. J Mol Cell Cardiol (2019) 136:27–41. doi: 10.1016/j.yjmcc.2019.09.001 31505198

[140] Jiang M Qi L Li L Wu Y Song D Li Y. Caspase-8: A key protein of cross-talk signal way in "PANoptosis" in cancer. Int J Cancer (2021) 149(7):1408–20. doi: 10.1002/ijc.33698 34028029

[141] Allan LA Clarke PR. Apoptosis and autophagy: Regulation of caspase-9 by phosphorylation. FEBS J (2009) 276(21):6063–73. doi: 10.1111/j.1742-4658.2009.07330.x 19788417

[142] Hilgendorf I Swirski FK Robbins CS. Monocyte fate in atherosclerosis. Arterioscler Thromb Vasc Biol (2015) 35(2):272–9. doi: 10.1161/ATVBAHA.114.303565 25538208

[143] Lancaster CE Ho CY Hipolito VEB Botelho RJ Terebiznik MR. Phagocytosis: what's on the menu? (1). Biochem Cell Biol (2019) 97(1):21–9. doi: 10.1139/bcb-2018-0008 29791809

[144] Pierce SK Morris JF Grusby MJ Kaumaya P Buskirk van A Srinivasan M. Antigen-presenting function of b lymphocytes. Immunol Rev (1988) 106:149–80. doi: 10.1111/j.1600-065X.1988.tb00778.x 3075588

[145] Costa-García M Ataya M Moraru M Vilches C López-Botet M Muntasell A. Human cytomegalovirus antigen presentation by HLA-DR+ NKG2C+ adaptive NK cells specifically activates polyfunctional effector memory CD4+ T lymphocytes. Front Immunol (2019) 10:687. doi: 10.3389/fimmu.2019.00687 31001281 PMC6456717

[146] Wang D Yuan F Wang L Wei W. Paeoniflorin inhibits function and down-regulates HLA-DR and CD80 expression of human peripheral blood monocytes stimulated by rhIL-1β. Int Immunopharmacol (2012) 14(2):172–8. doi: 10.1016/j.intimp.2012.07.005 22835428

[147] Kanter JE. Monocyte recruitment versus macrophage proliferation in atherosclerosis. Circ Res (2017) 121(10):1109–10. doi: 10.1161/CIRCRESAHA.117.311973 PMC5687308 29074524

[148] Lee GR. The balance of Th17 versus treg cells in autoimmunity. Int J Mol Sci (2018) 19(3). doi: 10.3390/ijms19030730 PMC5877591 29510522

[149] Taleb S Tedgui A. IL-17 in atherosclerosis: the good and the bad. Cardiovasc Res (2018) 114(1):7–9. doi: 10.1093/cvr/cvx225 29228116

[150] Li Q Liu Y Xia X Sun H Gao J Ren Q. Activation of macrophage TBK1-HIF-1α-mediated IL-17/IL-10 signaling by hyperglycemia aggravates the complexity of coronary atherosclerosis: An in vivo and in vitro study. FASEB J (2021) 35(5):e21609. doi: 10.1096/fj.202100086RR 33908659

[151] Radzyukevich YV Kosyakova NI Prokhorenko IR. Participation of monocyte subpopulations in progression of experimental endotoxemia (EE) and systemic inflammation. J Immunol Res (2021) 2021:1762584. doi: 10.1155/2021/1762584 33628841 PMC7895567

[152] Dai X Wang LW Jia XY Chang Y Wu HX Wang C. Paeoniflorin regulates the function of human peripheral blood mononuclear cells stimulated by rhIL-1β by up-regulating treg expression. Immunopharmacol Immunotoxicol (2015) 37(3):252–7. doi: 10.3109/08923973.2015.1026603 25986991

[153] Tabas I Bornfeldt KE. Macrophage phenotype and function in different stages of atherosclerosis. Circ Res (2016) 118(4):653–67. doi: 10.1161/CIRCRESAHA.115.306256 PMC4762068 26892964

[154] Orihuela R McPherson CA Harry GJ. Microglial M1/M2 polarization and metabolic states. Br J Pharmacol (2016) 173(4):649–65. doi: 10.1111/bph.13139 PMC4742299 25800044

[155] Sica A Mantovani A. Macrophage plasticity and polarization: in vivo veritas. J Clin Invest (2012) 122(3):787–95. doi: 10.1172/JCI59643 PMC3287223 22378047

[156] Udalova IA Mantovani A Feldmann M. Macrophage heterogeneity in the context of rheumatoid arthritis. Nat Rev Rheumatol (2016) 12(8):472–85. doi: 10.1038/nrrheum.2016.91 27383913

[157] Meraz MA White JM Sheehan KC Bach EA Rodig SJ Dighe AS. Targeted disruption of the Stat1 gene in mice reveals unexpected physiologic specificity in the JAK-STAT signaling pathway. Cell (1996) 84(3):431–42. doi: 10.1016/S0092-8674(00)81288-X 8608597

[158] Gong M Zhuo X Ma A. STAT6 upregulation promotes M2 macrophage polarization to suppress atherosclerosis. Med Sci Monit Basic Res (2017) 23:240–9. doi: 10.12659/MSMBR.904014 PMC5484610 28615615

[159] Wang D Yang F Shang W Zhao Z Shen J Cai H. Paeoniflorin-loaded pH-sensitive liposomes alleviate synovial inflammation by altering macrophage polarity via STAT signaling. Int Immunopharmacol (2021) 101(Pt A):108310. doi: 10.1016/j.intimp.2021.108310 34749294

[160] Zhai T Sun Y Li H Zhang J Huo R Li H. Unique immunomodulatory effect of paeoniflorin on type I and II macrophages activities. J Pharmacol Sci (2016) 130(3):143–50. doi: 10.1016/j.jphs.2015.12.007 26852260

[161] Shao YX Xu XX Li YY Qi XM Wang K Wu YG. Paeoniflorin inhibits high glucose-induced macrophage activation through TLR2-dependent signal pathways. J Ethnopharmacol (2016) 193:377–86. doi: 10.1016/j.jep.2016.08.035 27566204

[162] Shao YX Xu XX Wang K Qi XM Wu YG. Paeoniflorin attenuates incipient diabetic nephropathy in streptozotocin-induced mice by the suppression of the toll-like receptor-2 signaling pathway. Drug Des Devel Ther (2017) 11:3221–33. doi: 10.2147/DDDT.S149504 PMC5687495 29184392

[163] Li C Xu MM Wang K Adler AJ Vella AT Zhou B. Macrophage polarization and meta-inflammation. Transl Res (2018) 191:29–44. doi: 10.1016/j.trsl.2017.10.004 29154757 PMC5776711

[164] Cao L Yang K. Paeoniflorin attenuated TREM-1-Mediated inflammation in THP-1 cells. J Healthc Eng (2022) 2022:7051643. doi: 10.1155/2022/7051643 35480155 PMC9038380

[165] Fang Y Wu LC Ma K Pan G Yang S Zheng Y. Paeoniflorin alleviates lipopolysaccharide-induced disseminated intravascular coagulation by inhibiting inflammation and coagulation activation. Drug Dev Res (2020) 81(4):517–25. doi: 10.1002/ddr.21647 32065451

[166] Zhang J Dou W Zhang E Sun A Ding L Wei X. Paeoniflorin abrogates DSS-induced colitis via a TLR4-dependent pathway. Am J Physiol Gastrointest Liver Physiol (2014) 306(1):G27–36. doi: 10.1152/ajpgi.00465.2012 PMC3920084 24232001

[167] Wang QS Gao T Cui YL Gao LN Jiang HL. Comparative studies of paeoniflorin and albiflorin from paeonia lactiflora on anti-inflammatory activities. Pharm Biol (2014) 52(9):1189–95. doi: 10.3109/13880209.2014.880490 24646307

[168] Zhang T Zhu Q Shao Y Wang K Wu Y. Paeoniflorin prevents TLR2/4-mediated inflammation in type 2 diabetic nephropathy. Biosci Trends (2017) 11(3):308–18. doi: 10.5582/bst.2017.01104 28626209

[169] Liu Z Zhang J Jiang P Yin Z Liu Y Liu Y. Paeoniflorin inhibits the macrophage-related rosacea-like inflammatory reaction through the suppressor of cytokine signaling 3-apoptosis signal-regulating kinase 1-p38 pathway. Med (Baltimore) (2021) 100(3):e23986. doi: 10.1097/MD.0000000000023986 PMC7837818 33545988

[170] Sheng L Hu F Yu H Tao X Jia R Gu Y. Paeoniflorin inhibits ASK1-TF axis by up-regulating SOCS3 to alleviate radiation enteritis. Front Pharmacol (2022) 13:743708. doi: 10.3389/fphar.2022.743708 35359871 PMC8964139

[171] Jiang WL Chen XG Zhu HB Gao YB Tian JW Fu FH. Paeoniflorin inhibits systemic inflammation and improves survival in experimental sepsis. Basic Clin Pharmacol Toxicol (2009) 105(1):64–71. doi: 10.1111/j.1742-7843.2009.00415.x 19371254

[172] Seimon T Tabas I. Mechanisms and consequences of macrophage apoptosis in atherosclerosis. J Lipid Res (2009) 50 Suppl(Suppl):S382–7. doi: 10.1194/jlr.R800032-JLR200 PMC2674693 18953058

[173] Qin Z Wang PY Su DF Liu X. miRNA-124 in immune system and immune disorders. Front Immunol (2016) 7:406. doi: 10.3389/fimmu.2016.00406 27757114 PMC5047895

[174] Zhai C Cong H Hou K Hu Y Zhang J Zhang Y. Effects of miR-124-3p regulation of the p38MAPK signaling pathway via MEKK3 on apoptosis and proliferation of macrophages in mice with coronary atherosclerosis. Adv Clin Exp Med (2020) 29(7):803–12. doi: 10.17219/acem/121926 32750754

[175] Veremeyko T Siddiqui S Sotnikov I Yung A Ponomarev ED. IL-4/IL-13-dependent and independent expression of miR-124 and its contribution to M2 phenotype of monocytic cells in normal conditions and during allergic inflammation. PloS One (2013) 8(12):e81774. doi: 10.1371/journal.pone.0081774 24358127 PMC3864800

[176] Huang D Li Z Chen Y Fan Y Yu T. Paeoniflorin reduces the inflammatory response of THP-1 cells by up-regulating microRNA-124 : Paeoniflorin reduces the inflammatory response of THP-1 cells through microRNA-124. Genes Genomics (2021) 43(6):623–31. doi: 10.1007/s13258-021-01083-2 PMC8131308 33779948

[177] Shi J Gao W Shao F. Pyroptosis: Gasdermin-mediated programmed necrotic cell death. Trends Biochem Sci (2017) 42(4):245–54. doi: 10.1016/j.tibs.2016.10.004 27932073

[178] Jie F Xiao S Qiao Y You Y Feng Y Long Y. Kuijieling decoction suppresses NLRP3-mediated pyroptosis to alleviate inflammation and experimental colitis in vivo and in vitro. J Ethnopharmacol (2021) 264:113243. doi: 10.1016/j.jep.2020.113243 32781258

[179] Yu P Zhang X Liu N Tang L Peng C Chen X. Pyroptosis: mechanisms and diseases. Signal Transduct Target Ther (2021) 6(1):128. doi: 10.1038/s41392-021-00507-5 33776057 PMC8005494

[180] Xu L Wang H Yu QQ Ge JR Zhang XZ Mei D. The monomer derivative of paeoniflorin inhibits macrophage pyroptosis via regulating TLR4/ NLRP3/ GSDMD signaling pathway in adjuvant arthritis rats. Int Immunopharmacol (2021) 101(Pt A):108169. doi: 10.1016/j.intimp.2021.108169 34607227

[181] Barrett TJ. Macrophages in atherosclerosis regression. Arterioscler Thromb Vasc Biol (2020) 40(1):20–33. doi: 10.1161/ATVBAHA.119.312802 31722535 PMC6946104

[182] Zhang MH Feng L Zhu MM Gu JF Wu C Jia XB. Antioxidative and anti-inflammatory activities of paeoniflorin and oxypaeoniflora on AGEs-induced mesangial cell damage. Planta Med (2013) 79(14):1319–23. doi: 10.1055/s-0033-1350649 23881455

[183] Tsai JY Su KH Shyue SK Kou YR Yu YB Hsiao SH. EGb761 ameliorates the formation of foam cells by regulating the expression of SR-a and ABCA1: role of haem oxygenase-1. Cardiovasc Res (2010) 88(3):415–23. doi: 10.1093/cvr/cvq226 20615914

[184] Schmitz J Owyang A Oldham E Song Y Murphy E McClanahan TK. IL-33, an interleukin-1-like cytokine that signals via the IL-1 receptor-related protein ST2 and induces T helper type 2-associated cytokines. Immunity (2005) 23(5):479–90. doi: 10.1016/j.immuni.2005.09.015 16286016

[185] Moussion C Ortega N Girard JP. The IL-1-like cytokine IL-33 is constitutively expressed in the nucleus of endothelial cells and epithelial cells in vivo: a novel 'alarmin'? PloS One (2008) 3(10):e3331. doi: 10.1371/journal.pone.0003331 18836528 PMC2556082

[186] Aimo A Migliorini P Vergaro G Franzini M Passino C Maisel A. The IL-33/ST2 pathway, inflammation and atherosclerosis: Trigger and target? Int J Cardiol (2018) 267:188–92. doi: 10.1016/j.ijcard.2018.05.056 29793758

[187] Li W Tao W Chen J Zhai Y Yin N Wang Z. Paeoniflorin suppresses IL-33 production by macrophages. Immunopharmacol Immunotoxicol (2020) 42(3):286–93. doi: 10.1080/08923973.2020.1750628 32312124

[188] Kim ID Ha BJ. Paeoniflorin protects RAW 264.7 macrophages from LPS-induced cytotoxicity and genotoxicity. Toxicol In Vitro (2009) 23(6):1014–9. doi: 10.1016/j.tiv.2009.06.019 19540912

[189] Higashi Y Noma K Yoshizumi M Kihara Y. Endothelial function and oxidative stress in cardiovascular diseases. Circ J (2009) 73(3):411–8. doi: 10.1253/circj.CJ-08-1102 19194043

[190] Jackson SP. The growing complexity of platelet aggregation. Blood (2007) 109(12):5087–95. doi: 10.1182/blood-2006-12-027698 17311994

[191] Kroll MH Hellums JD McIntire LV Schafer AI Moake JL. Platelets and shear stress. Blood (1996) 88(5):1525–41. doi: 10.1182/blood.V88.5.1525.1525 8781407

[192] Nieswandt B Pleines I Bender M. Platelet adhesion and activation mechanisms in arterial thrombosis and ischaemic stroke. J Thromb Haemost (2011) 9 Suppl 1:92–104. doi: 10.1111/j.1538-7836.2011.04361.x 21781245

[193] Li Z Delaney MK O'Brien KA Du X. Signaling during platelet adhesion and activation. Arterioscler Thromb Vasc Biol (2010) 30(12):2341–9. doi: 10.1161/ATVBAHA.110.207522 PMC3085271 21071698

[194] Zhang GM Zhang W Zhang GM. Age-specific reference intervals for PT, aPTT, fibrinogen and thrombin time for parturient women. Thromb Haemost (2019) 119(6):894–8. doi: 10.1055/s-0039-1683932 30934105

[195] Ngo T Kim K Bian Y Noh H Lim KM Chung JH. Antithrombotic effects of paeoniflorin from paeonia suffruticosa by selective inhibition on shear stress-induced platelet aggregation. Int J Mol Sci (2019) 20(20). doi: 10.3390/ijms20205040 PMC6834133 31614534

[196] Ye S Mao B Yang L Fu W Hou J. Thrombosis recanalization by paeoniflorin through the upregulation of urokinasetype plasminogen activator via the MAPK signaling pathway. Mol Med Rep (2016) 13(6):4593–8. doi: 10.3892/mmr.2016.5146 PMC4878539 27082639

[197] Ye J Duan H Yang X Yan W Zheng X. Anti-thrombosis effect of paeoniflorin: evaluated in a photochemical reaction thrombosis model in vivo. Planta Med (2001) 67(8):766–7. doi: 10.1055/s-2001-18364 11731926

[198] Zhu M Tang Y Duan JA Guo J Guo S Su S. Roles of paeoniflorin and senkyunolide I in SiWu decoction on antiplatelet and anticoagulation activities. J Sep Sci (2010) 33(21):3335–40. doi: 10.1002/jssc.201000340 20878657

[199] Xie P Cui L Shan Y Kang WY. Antithrombotic effect and mechanism of radix paeoniae rubra. BioMed Res Int 2017 (2017) p:9475074. doi: 10.1155/2017/9475074 PMC5337344 28299338

[200] da Silva EZ Jamur MC Oliver C. Mast cell function: a new vision of an old cell. J Histochem Cytochem (2014) 62(10):698–738. doi: 10.1369/0022155414545334 25062998 PMC4230976

[201] Bot I Biessen EA. Mast cells in atherosclerosis. Thromb Haemost (2011) 106(5):820–6. doi: 10.1160/th11-05-0291 21866302

[202] Bot I Shi GP Kovanen PT. Mast cells as effectors in atherosclerosis. Arterioscler Thromb Vasc Biol (2015) 35(2):265–71. doi: 10.1161/ATVBAHA.114.303570 PMC4304944 25104798

[203] Oettgen HC Burton OT. IgE and mast cells: The endogenous adjuvant. Adv Immunol (2015) 127:203–56. doi: 10.1016/bs.ai.2015.03.001 26073985

[204] Zhang Y Hu S Ge S Wang J He L. Paeoniflorin inhibits IgE-mediated allergic reactions by suppressing the degranulation of mast cells though binding with FcRI alpha subunits. Eur J Pharmacol (2020) 886:173415. doi: 10.1016/j.ejphar.2020.173415 32771669

[205] Wang G Cheng N. Paeoniflorin inhibits mast cell-mediated allergic inflammation in allergic rhinitis. J Cell Biochem (2018) 119(10):8636–42. doi: 10.1002/jcb.27135 30076630

[206] Zhao Y Li X Chu J Shao Y Sun Y Zhang Y. Inhibitory effect of paeoniflorin on IgE-dependent and IgE-independent mast cell degranulation in vitro and vivo. Food Funct (2021) 12(16):7448–68. doi: 10.1039/D1FO01421H 34195738

[207] Gould RG. Lipid metabolism and atherosclerosis. Am J Med (1951) 11(2):209–27. doi: 10.1016/0002-9343(51)90107-6 14856990

[208] Zhang S Hong F Ma C Yang S. Hepatic lipid metabolism disorder and atherosclerosis. Endocr Metab Immune Disord Drug Targets (2022) 22(6):590–600. doi: 10.2174/1871530322666211220110810 34931971

[209] Khetarpal SA Schjoldager KT Christoffersen C Raghavan A Edmondson AC Reutter HM. Loss of function of GALNT2 lowers high-density lipoproteins in humans, nonhuman primates, and rodents. Cell Metab (2016) 24(2):234–45. doi: 10.1016/j.cmet.2016.07.012 PMC5663192 27508872

[210] Xiao HB Wang JY Sun ZL. ANGPTL3 is part of the machinery causing dyslipidemia majorily via LPL inhibition in mastitis mice. Exp Mol Pathol (2017) 103(3):242–8. doi: 10.1016/j.yexmp.2017.11.003 29104012

[211] Olvera-Sandoval C Betanzos-Cabrera G Casillas-Peñuelas R Quintanar JL. Changes in body composition and mRNA expression of ghrelin and lipoprotein lipase in rats treated with leuprolide acetate, a GnRH agonist. Exp Ther Med (2018) 15(1):592–8. doi: 10.3892/etm.2017.5352 PMC5729720 29250162

[212] Yang HO Ko WK Kim JY Ro HS. Paeoniflorin: an antihyperlipidemic agent from paeonia lactiflora. Fitoterapia (2004) 75(1):45–9. doi: 10.1016/j.fitote.2003.08.016 14693219

[213] Xiao HB Liang L Luo ZF Sun ZL. Paeoniflorin regulates GALNT2-ANGPTL3-LPL pathway to attenuate dyslipidemia in mice. Eur J Pharmacol (2018) 836:122–8. doi: 10.1016/j.ejphar.2018.08.006 30096295

[214] Li YC Qiao JY Wang BY Bai M Shen JD Cheng YX. Paeoniflorin ameliorates fructose-induced insulin resistance and hepatic steatosis by activating LKB1/AMPK and AKT pathways. Nutrients (2018) 10(8). doi: 10.3390/nu10081024 PMC6116094 30081580

[215] Kikuchi T Orihara K Oikawa F Han SI Kuba M Okuda K. Intestinal CREBH overexpression prevents high-cholesterol diet-induced hypercholesterolemia by reducing Npc1l1 expression. Mol Metab (2016) 5(11):1092–102. doi: 10.1016/j.molmet.2016.09.004 PMC5081412 27818935

[216] Osaki Y Nakagawa Y Miyahara S Iwasaki H Ishii A Matsuzaka T. Skeletal muscle-specific HMG-CoA reductase knockout mice exhibit rhabdomyolysis: A model for statin-induced myopathy. Biochem Biophys Res Commun (2015) 466(3):536–40. doi: 10.1016/j.bbrc.2015.09.065 26381177

[217] Goldstein JL Brown MS. The LDL receptor. Arterioscler Thromb Vasc Biol (2009) 29(4):431–8. doi: 10.1161/ATVBAHA.108.179564 PMC2740366 19299327

[218] Pyper SR Viswakarma N Yu S Reddy JK. PPARalpha: energy combustion, hypolipidemia, inflammation and cancer. Nucl Recept Signal (2010) 8:e002. doi: 10.1621/nrs.08002 20414453 PMC2858266

[219] Wang Y Han Y Chai F Xiang H Huang T Kou S. The antihypercholesterolemic effect of columbamine from rhizoma coptidis in HFHC-diet induced hamsters through HNF-4α/FTF-mediated CYP7A1 activation. Fitoterapia (2016) 115:111–21. doi: 10.1016/j.fitote.2016.09.019 27713083

[220] Hu H Zhu Q Su J Wu Y Zhu Y Wang Y. Effects of an enriched extract of paeoniflorin, a monoterpene glycoside used in Chinese herbal medicine, on cholesterol metabolism in a hyperlipidemic rat model. Med Sci Monit (2017) 23:3412–27. doi: 10.12659/MSM.905544 PMC5524283 28706181

[221] Kidani Y Bensinger SJ. Liver X receptor and peroxisome proliferator-activated receptor as integrators of lipid homeostasis and immunity. Immunol Rev (2012) 249(1):72–83. doi: 10.1111/j.1600-065X.2012.01153.x 22889216 PMC4007066

[222] Afonso Mda S Castilho G Lavrador MS Passarelli M Nakandakare ER Lottenberg SA. The impact of dietary fatty acids on macrophage cholesterol homeostasis. J Nutr Biochem (2014) 25(2):95–103. doi: 10.1016/j.jnutbio.2013.10.001 24445035

[223] Xiao L Wang J Jiang M Xie W Zhai Y. The emerging role of constitutive androstane receptor and its cross talk with liver X receptors and peroxisome proliferator-activated receptor a in lipid metabolism. Vitam Horm (2013) 91:243–58. doi: 10.1016/B978-0-12-407766-9.00010-9 23374719

[224] Zhang L Yang B Yu B. Paeoniflorin protects against nonalcoholic fatty liver disease induced by a high-fat diet in mice. Biol Pharm Bull (2015) 38(7):1005–11. doi: 10.1248/bpb.b14-00892 25972092

[225] Daina A Michielin O Zoete V. SwissADME: a free web tool to evaluate pharmacokinetics, drug-likeness and medicinal chemistry friendliness of small molecules. Sci Rep (2017) 7:42717. doi: 10.1038/srep42717 28256516 PMC5335600

[226] Wang L Phan DD Zhang J Ong PS Thuya WL Soo R. Anticancer properties of nimbolide and pharmacokinetic considerations to accelerate its development. Oncotarget (2016) 7(28):44790–802. doi: 10.18632/oncotarget.8316 PMC5190135 27027349

[227] Yang XZ Wei W. CP-25, a compound derived from paeoniflorin: research advance on its pharmacological actions and mechanisms in the treatment of inflammation and immune diseases. Acta Pharmacol Sin (2020) 41(11):1387–94. doi: 10.1038/s41401-020-00510-6 PMC7656585 32884075

[228] Qian J Cheng W Zhang C Hong L Chen W Li G. Preparation, physicochemical characterization and pharmacokinetics of paeoniflorin-phospholipid complex. BioMed Mater Eng (2019) 30(1):11–22. doi: 10.3233/BME-181029 30530955

[229] Shen C Shen B Zhu J Wang J Yuan H Li X. Glycyrrhizic acid-based self-assembled micelles for improving oral bioavailability of paeoniflorin. Drug Dev Ind Pharm (2021) 47(2):207–14. doi: 10.1080/03639045.2020.1862178 33305640

[230] Bonaca MP Hamburg NM Creager MA. Contemporary medical management of peripheral artery disease. Circ Res (2021) 128(12):1868–84. doi: 10.1161/CIRCRESAHA.121.318258 34110910

[231] Siasos G Tsigkou V Kokkou E Oikonomou E Vavuranakis M Vlachopoulos C. Smoking and atherosclerosis: mechanisms of disease and new therapeutic approaches. Curr Med Chem (2014) 21(34):3936–48. doi: 10.2174/092986732134141015161539 25174928

[232] Psaltopoulou T Hatzis G Papageorgiou N Androulakis E Briasoulis A Tousoulis D. Socioeconomic status and risk factors for cardiovascular disease: Impact of dietary mediators. Hellenic J Cardiol (2017) 58(1):32–42. doi: 10.1016/j.hjc.2017.01.022 28161284

[233] Lovren F Teoh H Verma S. Obesity and atherosclerosis: mechanistic insights. Can J Cardiol (2015) 31(2):177–83. doi: 10.1016/j.cjca.2014.11.031 25661552

[234] Laufs U Wassmann S Czech T Münzel T Eisenhauer M Böhm M. Physical inactivity increases oxidative stress, endothelial dysfunction, and atherosclerosis. Arterioscler Thromb Vasc Biol (2005) 25(4):809–14. doi: 10.1161/01.ATV.0000158311.24443.af 15692095

